# Decoding Distinct Ganglioside Patterns of Native and Differentiated Mesenchymal Stem Cells by a Novel Glycolipidomics Profiling Strategy

**DOI:** 10.1021/jacsau.2c00230

**Published:** 2022-10-25

**Authors:** Katharina Hohenwallner, Nina Troppmair, Lisa Panzenboeck, Cornelia Kasper, Yasin El Abiead, Gunda Koellensperger, Leonida M. Lamp, Jürgen Hartler, Dominik Egger, Evelyn Rampler

**Affiliations:** † Department of Analytical Chemistry, Faculty of Chemistry, University of Vienna, Vienna 1090, Austria; ‡ Vienna Doctoral School in Chemistry (DoSChem), University of Vienna, Vienna 1090, Austria; § Institute of Cell and Tissue Culture Technologies, University of Natural Resources and Life Sciences, Vienna 1190, Austria; ∥ Institute of Pharmaceutical Sciences, University of Graz, Graz 8010, Austria; ⊥ Field of Excellence BioHealth − University of Graz, Graz 8010, Austria

**Keywords:** ganglioside, mesenchymal stem cells, differentiation, human, glycolipidomics, mass spectrometry, LC−MS^n^, automated annotation

## Abstract

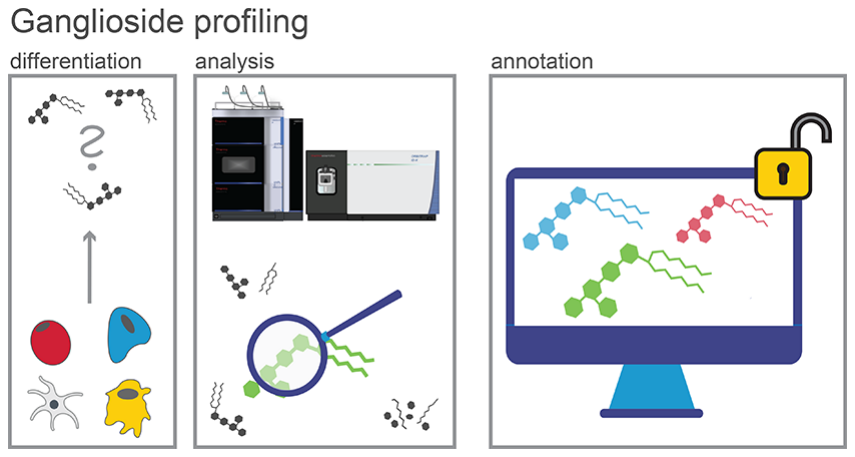

Gangliosides are an indispensable glycolipid class concentrated on cell surfaces with a critical role in stem cell differentiation. Nonetheless, owing to the lack of suitable methods for scalable analysis covering the full scope of ganglioside molecular diversity, their mechanistic properties in signaling and differentiation remain undiscovered to a large extent. This work introduces a sensitive and comprehensive ganglioside assay based on liquid chromatography, high-resolution mass spectrometry, and multistage fragmentation. Complemented by an open-source data evaluation workflow, we provide automated in-depth lipid species-level and molecular species-level annotation based on decision rule sets for all major ganglioside classes. Compared to conventional state-of-the-art methods, the presented ganglioside assay offers (1) increased sensitivity, (2) superior structural elucidation, and (3) the possibility to detect novel ganglioside species. A major reason for the highly improved sensitivity is the optimized spectral readout based on the unique capability of two parallelizable mass analyzers for multistage fragmentation. We demonstrated the high-throughput universal capability of our novel analytical strategy by identifying 254 ganglioside species. As a proof of concept, 137 unique gangliosides were annotated in native and differentiated human mesenchymal stem cells including 78 potential cell-state-specific markers and 38 previously unreported gangliosides. A general increase of the ganglioside numbers upon differentiation was observed as well as cell-state-specific clustering based on the ganglioside species patterns. The combination of the developed glycolipidomics assay with the extended automated annotation tool enables comprehensive in-depth ganglioside characterization as shown on biological samples of interest. Our results suggest ganglioside patterns as a promising quality control tool for stem cells and their differentiation products. Additionally, we believe that our analytical workflow paves the way for probing glycolipid-based biochemical processes shedding light on the enigmatic processes of gangliosides and glycolipids in general.

## Introduction

Gangliosides play a crucial structural role in the curvature of the plasma membrane. They are involved in many critical biological pathways related to cell–cell communication, cellular growth, host–pathogen interaction, and signal transduction. Gangliosides protrude from eukaryotic cell surfaces presenting their mobile hydrophilic glycan moiety to the outside, whereas the lipid moiety is cohesively integrated into the hydrophobic plasma membrane. The glycan moiety of gangliosides belongs to the glycocalyx, a dense gel-like matrix surrounding the plasma membrane of a cell, also known as “sweet husk”, which is active in various cellular processes.^1^ Gangliosides can serve as ligands and modulate the activity of membrane proteins depending on both the oligosaccharide head group and the ceramide anchor. In the literature, there are several different chemical definitions for gangliosides depending on the sugar moieties and branching in the glycan head group and their biosynthetic routes.^2^ In this work, we refer to gangliosides as acidic glycosphingolipids containing at least one sialic acid as defined by the comprehensive LIPID MAPS structure database.^3^ The highest concentrations of gangliosides are found in the central nervous system,^4^ and they are involved in several memory-related diseases, including Alzheimer’s, Parkinson’s and Huntington’s disease, AIDS-related dementia, and cancer.^5^ Recently, it was observed that gangliosides facilitate viral entry of SARS-CoV-2, highlighting their crucial role in interactions with the plasma membrane.^6^ In addition to their essential disease-related functions, ganglioside patterns vary with development stage and age. Dramatic changes in expression levels of gangliosides were observed during neurodevelopment,^4,7^ ranging from the expression of simple gangliosides with low numbers of sugars attached, e.g., GM3 and GD2 in early stages, to more complex gangliosides with higher sugar content in later developmental stages, particularly GM1, GD1a, GD1b, and GT1b.^4^ The strong influence of ganglioside composition in neurodevelopment was also expected in stem cell development. This hypothesis triggered investigations toward ganglioside biomarkers in differentiation processes.^8,9^ During human embryonic stem cell differentiation, a switch in the core structures of glycosphingolipids globo- and lacto- to ganglio-series was observed,^10^ leading to distinct alterations of specific glycosphingolipids.^11^

Mesenchymal stem/stromal cells (MSCs) comprise a heterogeneous cell population of nonhematopoietic stem cells, which are prevalently isolated from adipose tissue,^12^ bone marrow,^13^ or birth-associated tissues and fluids.^14,15^ They display a high proliferation and differentiation capacity and immunomodulatory effects on the innate and adaptive immune system,^16^ and anti-inflammatory and trophic effects on neighboring cells^17^ are observed. MSCs represent outstanding candidates for cell-based therapies and regenerative medicine applications, and their characterization is based on their surface profile expression and differentiation capacity.^18^ To enhance the quality control of cell-based therapeutic products, additional indicators are required to monitor and define the stem cell phenotype during *ex vivo* culture. Gangliosides are interesting marker candidates for cell and lineage-specific differentiation as they were found to be expressed in umbilical cord- and bone marrow-derived MSCs^19−21^ as well as during neural^8^ and osteogenic^9,11,22^ differentiation of MSCs. In accordance with these earlier studies, we recently found gangliosides to be upregulated in adipocytes compared to their human MSC progenitors.^23^ Gangliosides show huge structural variations in the glycan and lipid part depending on the cell type and state.^24,25^ As the ceramide structure and number of sialic acids or other sugar parts lead to changed properties, these structural fluctuations influence the membrane surrounding glycocalyx and cell signaling and development cascades. Gangliosides expressed in pluripotent, multipotent, and cancer stem cells have been traditionally identified by biochemical and immunological analysis.^8,20,26^ Although several fundamental studies indicated that gangliosides could be markers either for cell lineage, cell state, or function in different biological contexts,^9,20,27^ utilization of this observation failed so far, in part due to the lack of scalable, standardized methods enabling species-level assessment. Up to now, a comprehensive analysis of the expression pattern of gangliosides in native and differentiated MSCs is missing, making them an ideal test case for our study. Ganglioside analysis is extremely challenging since (1) the glycan and lipid part exhibit highly converse chemical properties, (2) targeted extraction protocols are needed, (3) only a few standards are available, and (4) suitable glycolipid databases are still absent. Gangliosides exhibit amphiphilic properties since they consist of a sugar head group linked to a lipid subunit. Thus, they are neither fully covered by common glycomics nor lipidomics analytical workflows. To fulfill the glycolipid function, both structural subunits are equally important (1) with the carbohydrate portion being responsible for the molecular recognition on the outer cell membrane and (2) the lipid portion being essential as hydrophobic anchor with cell-specific function potentially changing the carbohydrate orientation on the cell surface.^28^ Specialized glycolipidomics analytical workflows are required to bridge the gap between glycomics and lipidomics, unravel the complex glycolipid biology, and decipher additional structural information. As a matter of fact, such glycolipidomics strategies have to deal with the complexity of two extremely heterogeneous classes: glycans and lipids. The theoretical number of glycan and nonsaccharide permutations reaches almost Avogadro’s number,^29,30^ raising the group of glycolipids among the most complex biomolecules from a combinatorial perspective. State-of-the-art approaches, including classical immunological, biochemical, or thin-layer chromatography methods, entirely rely on class-specific ganglioside and glycolipid detection.^31^ For species characterization, they lack sensitivity or fail to provide detailed structural information, including the saccharide core (sialylation degree, anomers, branching) and the ceramide backbone (fatty acid, long-chain base, double bond position, and hydroxylation degree). Along the emergence of mass spectrometry-driven lipidomics, tailored ganglioside LC–MS workflows were proposed offering the possibility to collect both lipid and glycan information;^7,31−33^ however, most of the available workflows are still unable to resolve the molecular lipid species level. Predominantly, reversed-phase chromatography was reported as the method of choice,^7,31^ but hydrophilic interaction chromatography was also used for ganglioside class separation.^30^ More than a decade ago, a seminal review pointed out three methodological milestones required to remove the obstacles for glyco(sphingo)lipidomics research: (1) automated online interfacing such as LC or CE with ESI-MS, (2) ion trap applications with MS^n^ capabilities, and (3) computational bioinformatics offering automated species annotation of spectra.^34^ While there have been enormous advances regarding online class-specific separations prior to the mass spectrometric dimension, involving both high throughput UPLC and ion mobility, the latter two did not reach a satisfactory level yet. Despite the increased availability of sophisticated analytical workflows, the gangliosidome and glycol(sphingo)lipidome analysis remains a challenging task, typically conducted only by a few experts trained to manage the tedious and complex manual annotation of fragmentation spectra.

In this work, we implemented a comprehensive and sensitive large-scale profiling strategy for gangliosides for the first time. The workflow is based on an online reversed-phase high-resolution MS^n^ ganglioside assay to analyze intact glycosphingolipids and annotate them up to the level of functional groups.^35^ Using this novel LC–MS^n^ analytical strategy, all prevalent ganglioside classes (GM1, GM2, GM3, GM4, GD1, GD2, GD3, GT1, GT2, GT3, GQ1, GP1, detailed information regarding their structure can be found in Figure S1) are covered over a concentration range of four orders of magnitude. Species annotation is based on platform-independent decision rules^36,37^ reflecting information derived from the literature^31,38−40^ and experimentally obtained information. Normalization was based on a commercially available isotopically labeled standard, further allowing an estimation of present concentration ranges in a proof-of principle study based on biological samples of interest. By applying this strategy to the analysis of MSCs induced toward differentiation in chondrogenic, adipogenic, and osteogenic lineages, we revealed the highest number of gangliosides reported in a single study so far.^7,25,32^ Moreover, we found hints pointing toward the capability of gangliosides as cell differentiation markers for mesenchymal stem cells, highlighting the potential of our novel glycolipidomics assay.

## Results

### Surface Marker Expression and Differentiation of Human MSCs

**Figure 1:**
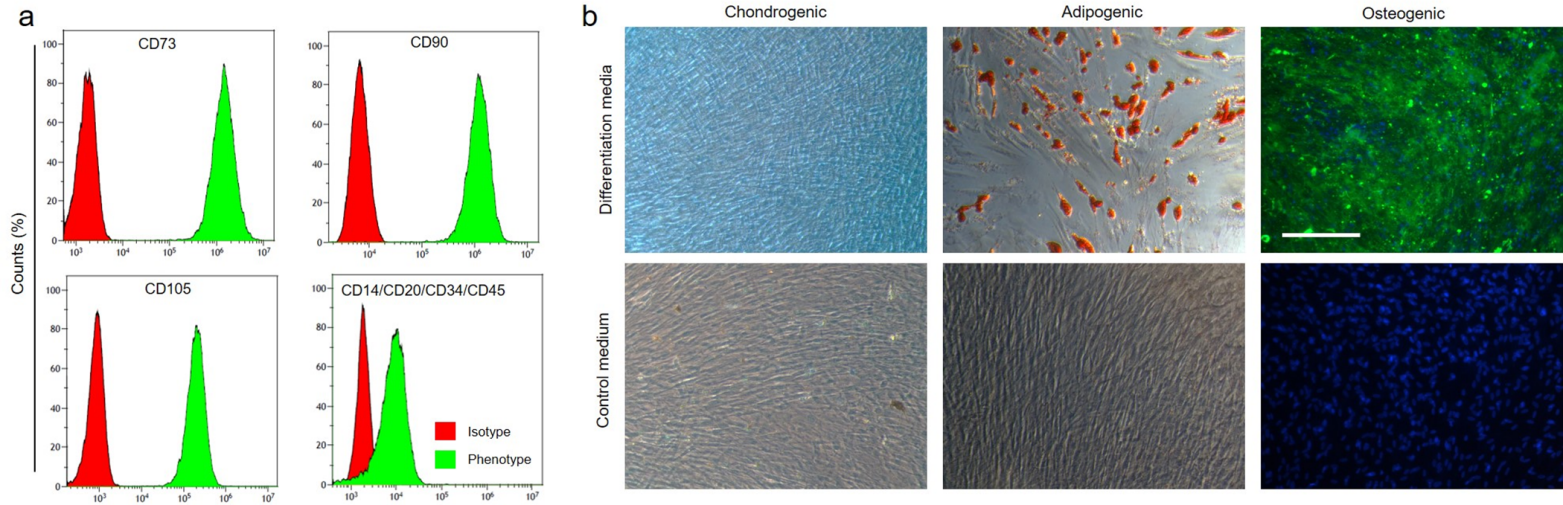
Characterization of human adipose-derived mesenchymal stem cells. (a) Flow cytometry histograms of adipose-derived MSCs stained for the respective surface markers after isolation in passage 2. (b) Micrographs of MSCs cultured in chondrogenic, adipogenic, and osteogenic medium for 21 days and stained for glycosaminoglycans by alcian blue (chondrogenic, blue), lipid vacuoles by Oil Red O (adipogenic, red), or calcium deposition by calcein (osteogenic, green; counterstain with DAPI for cell nuclei, blue), respectively. Standard culture medium without differentiation supplements served as control. The scale bar represents 250 μm.

MSCs are characterized by the ability to differentiate toward the adipogenic, chondrogenic, and osteogenic lineage and the expression of several surface markers.^18^ Several fundamental studies indicated that gangliosides are promising additional surface marker candidates either for cell lineage, cell state, or function in different biological contexts.^9,20,27^ A detailed ganglioside characterization is missing in native and differentiated MSCs; hence, we performed in-depth structural profiling of gangliosides in MSCs and differentiated cell lineages in our study. To confirm the identity of the adipose tissue-derived cells, the expression of several surface markers was assessed via antibody staining and flow cytometry. Conform to the minimal criteria of MSCs, we found cluster of differentitation (CD) 73, CD90, and CD105 to be expressed and CD14, CD20, CD34, and CD45 to be absent (Figure 1a). The MSCs were differentiated toward the chondrogenic, adipogenic, and osteogenic lineage using suitable media conditions. Histological staining confirmed the secretion of glycosaminoglycans in the chondrogenic samples, the formation of lipid vacuoles in the adipogenic samples, and the presence of calcium in the extracellular matrix of osteogenic samples that were cultured 21 days in the respective differentiation media (Figure 1b).

### Development of an Automated RP-HRMS^n^ Ganglioside Profiling Assay

**Figure 2:**
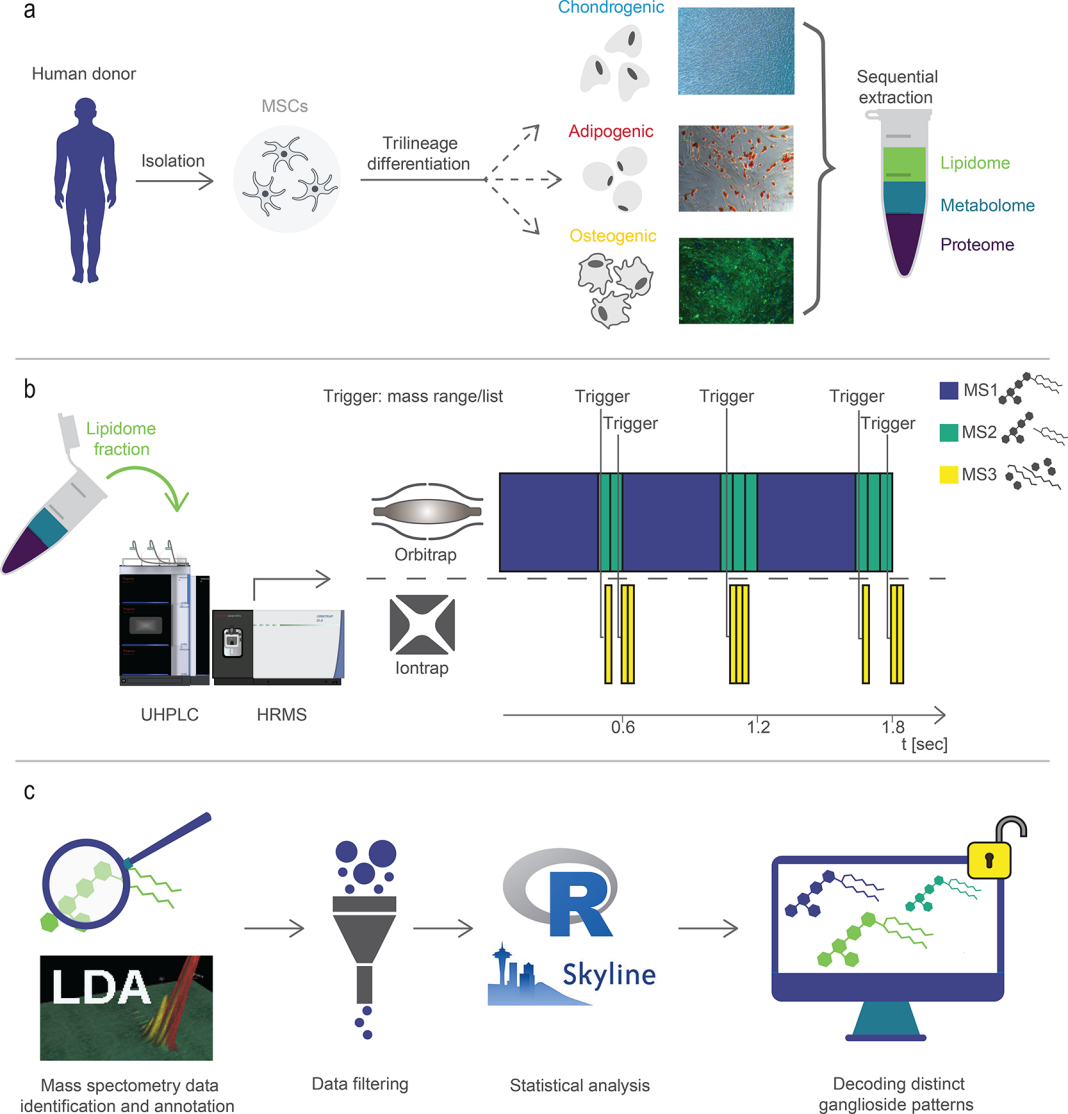
Developed glycolipidomics workflow at the example of mesenchymal stem cells. (a) Human MSCs were collected from surgical procedures and differentiated toward the adipogenic, chondrogenic, and osteogenic lineage. The lipid fraction was extracted and (b) analyzed via RP-HRMS^n^ using multistage fragmentation with two mass analyzers used in parallel for in-depth characterization of the gangliosides. (c) Data analysis was performed using an open-source workflow to decode distinct ganglioside patterns in native or differentiated MSCs.

**Figure 3:**
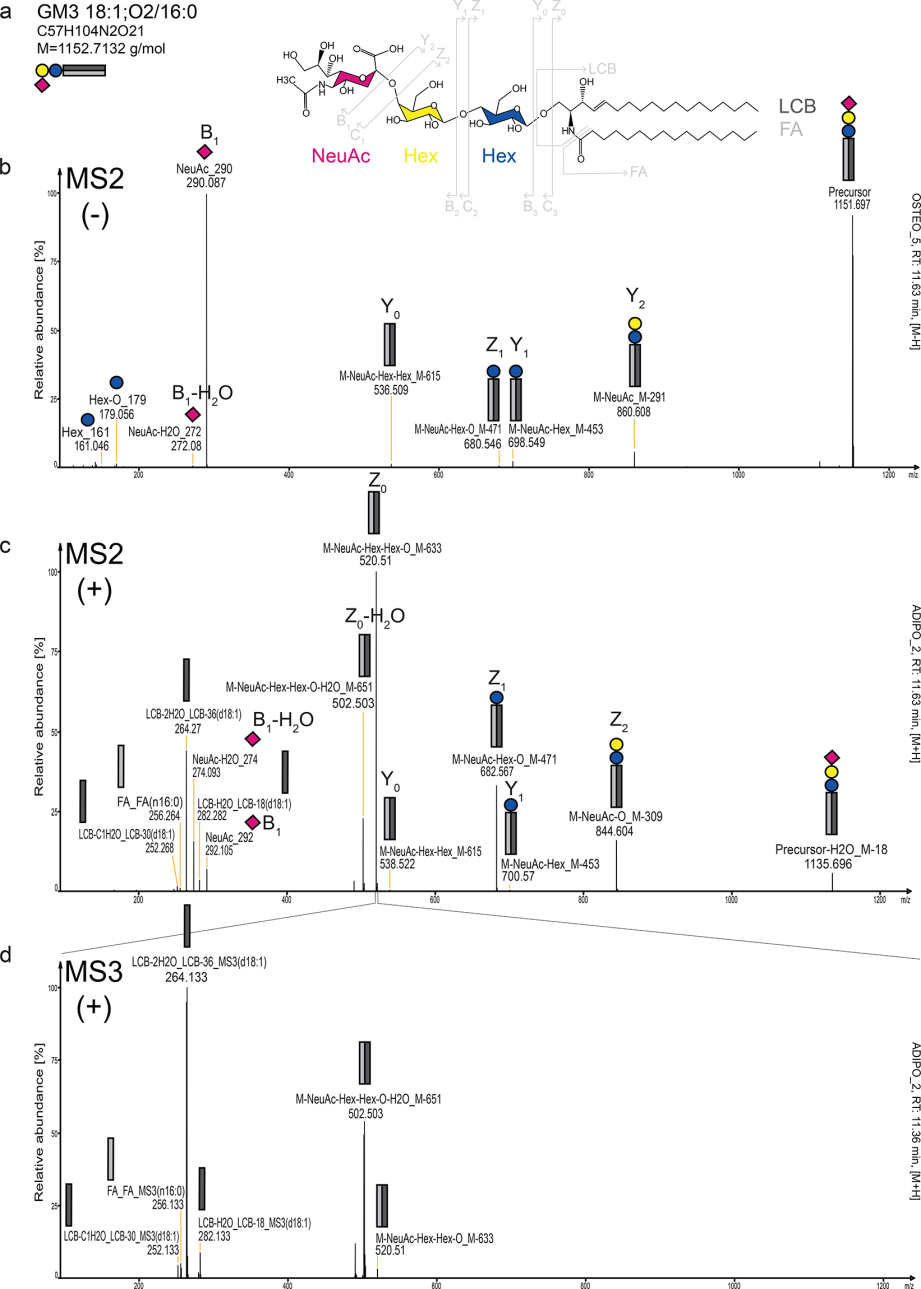
Molecular species ganglioside annotation by high-qualitiy spectra produced by the presented workflow. (a) Gangliosides have a lipid moiety containing a long-chain base (LCB) and fatty acid (FA), shown as gray rectangles. The glycan moiety (represented as blue and yellow circles) can vary in the number of hexoses (Hex, galactose and glucose; cannot be distinguished by fragmentation spectra) and in the number of sialic acids (NeuAc, pink diamond, see Figure S1 for more details). Spectral data of GM3 18:1;O2/16:0 in negative (b MS2) and positive (c MS2 and d MS3) ionization mode, respectively. In negative ionization mode, the fragments of the sugar part can be seen (Hex, NeuAc), as well as the intact precursor. In positive ion mode, the composition of the ceramide part can be resolved.

A novel ganglioside assay workflow has been developed, enabling in-depth structural analysis and automated annotation of gangliosides (Figure 2). We bridge lipid and glycan analysis workflows by combining (1) an extraction protocol on methyl *tert*-butyl ether,^41^ (2) reversed-phase high-resolution mass spectrometry and multi-stage fragmentation (RP-HRMS^n^), taking advantage of two parallel mass analyzers (Orbitrap, Ion Trap), and (3) a newly developed automated annotation workflow based on the open-source software Lipid Data Analyzer^36^ (LDA), followed by (4) applying strict filter criteria by an in-house developed R Script. Gangliosides were extracted from undifferentiated MSCs and differentiated toward adipogenic, chondrogenic, and osteogenic cells. Concentrations were determined by protein content normalization and normalization based on a deuterated internal standard (d5 GM1 36:1;O2). Reversed-phase ultrahigh-performance liquid chromatography was performed to separate the different ganglioside species based on their hydrophobic interaction with a C18 stationary phase. A standard acetonitrile isopropanol gradient^42^ was used to elute the gangliosides from the column, followed by lipid ionization in the heated electrospray source of the high-resolution mass spectrometer. In state-of-the-art HRMS workflows, glycosidic fragments are observed in negative mode only, whereas the ceramide part is monitored in positive mode only. In our workflow, the two mass analyzers (Orbitrap and Ion Trap) are used in parallel for multistage fragmentation (Figure 2b). By acquiring positive and negative ion mode data, we obtained fragments providing structural details for both glycosidic and lipid moieties (see Figure 3). The combination of MS2 and MS3 fragmentation permitted the structural characterization up to the lipid (molecular) species level^43,44^ of all major ganglioside classes present in native MSCs, three different cell lineages and ganglioside standard mixtures. Acquisition time optimization of both mass analyzers used in parallel toward higher information content highly improved the structural sensitity of our method (Figure 2).

Automated ganglioside annotation was performed based on in-house developed decision rule sets for the freely available software LDA optimized for sphingolipids LDA.^36^ To ensure annotation quality, we designated fragments specific for the different substructures of the corresponding ganglioside classes, which had to be detected. For this purpose, we selected one mandatory MS2 fragment followed by MS^n^ fragment assignment based on rules for the glycan (e.g., number of sialic acids) and lipid part (long-chain base (LCB) and fatty acid (FA) moiety, hydroxylation state) for all ganglioside classes detected. As an example, the fragmentation rules for the ganglioside class GM3 (neg mode, [M-H] adduct) can be found in Table S5. A simplified overview of the final glyoclipidomics profiling workflow can be found in Figure 2.

**Figure 4:**
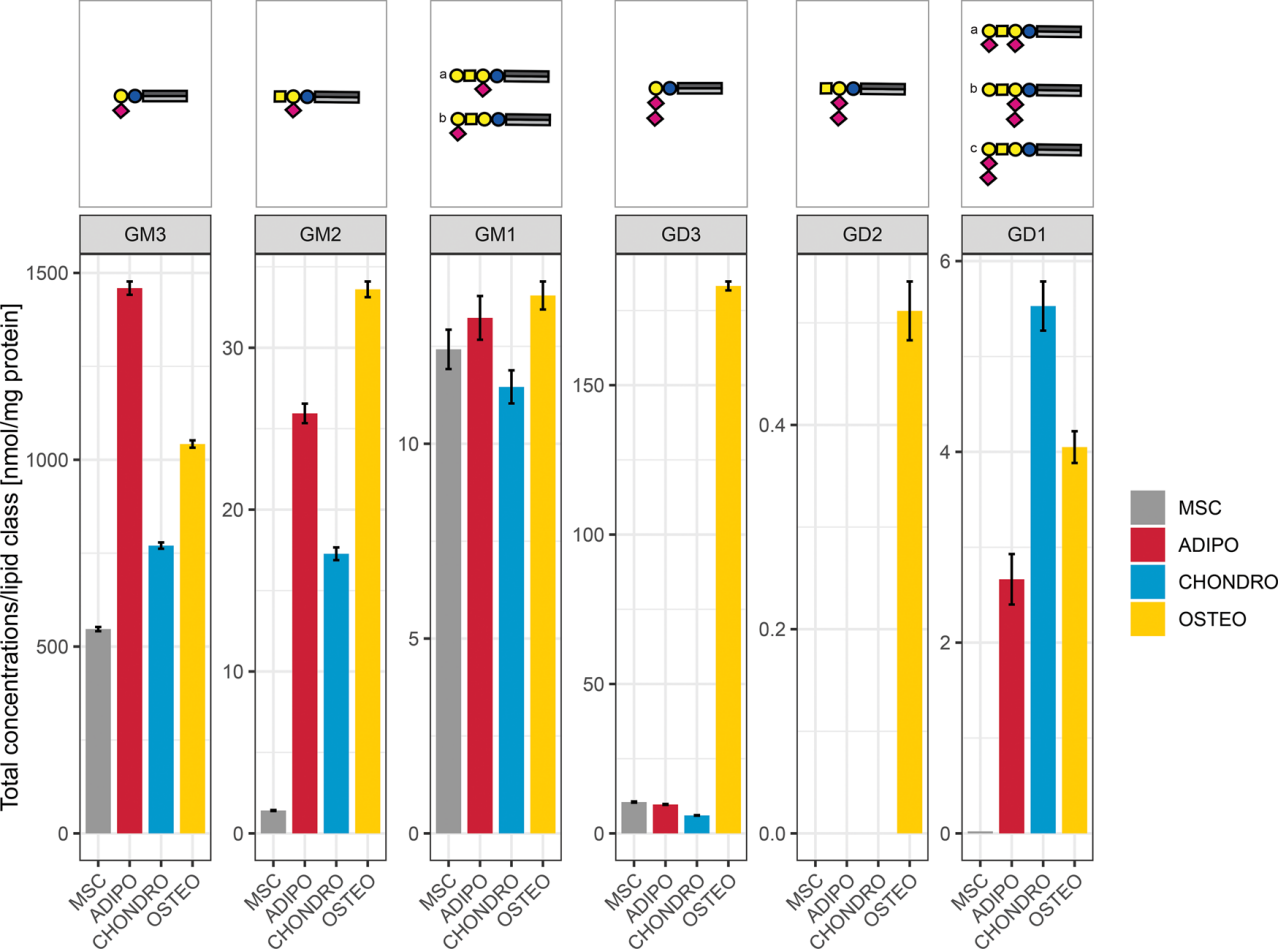
Lipid class overview according to detected sialic acid length in MSC and differentiated cell lineages. Six ganglioside classes are quantified in the different sample groups. The sum of adducts was calculated in negative ion mode, normalized to the internal standard d5 GM1 18:1;O2/18:0 and the protein content. Different glycan head group isomers are summarized for the ganglioside classes (for more information regarding the structure see Figure S1).

The presented glycolipidomics profiling strategy permits the detection of several hundred gangliosides in parallel using a combination of chromatographic separation, high-resolution mass spectrometry, and multistage fragmentation. In the analyzed samples and standards, we were able to identify 254 unique gangliosides of the classes (Figure S1) GM1, GM2, GM3, GM4, GD1, GD2, GD3, GT1, GT3, and GQ1 (Table S2). MS2 fragmentation was a prerequisite for the performed ganglioside annotation. Interestingly, 45% of all unique gangliosides had corresponding MS3 spectra in one of the samples or standards, highlighting the broad access to MS3 spectra, covering species of high and medium abundance. The combination of retention time and MS2 and MS3 fragment information was used by the LDA to automatically annotate the ganglioside species level (e.g., GM3 36:1;O2). The molecular lipid species level (e.g., GM3 18:1;O2/18:0) was also assigned by LDA if both lipid chains were annotated by MS2 or MS3 fragments in one ion mode (either positive or negative). In order to account for potential adduct ionization differences in the respective ganglioside classes, we used the sum of quantities from all individual adduct identifications as a quantitative measure for each identified species. The annotation was confirmed by applying additional automated filtering steps (see extended methods in the Supporting Information for more information) and retention time matching to commercially available ganglioside standards (total ganglioside mixtures, ganglioside classes, and deuterated single standard). The quality of the performed automated annotation step corresponded to category D meaning lipid species or molecular lipid species levels were determined.^35^ Despite a high degree of automation offered by the presented ganglioside annotation strategy, it has to be noted that LCB and FA chain assignment, necessary to determine molecular lipid species levels, needs to be confirmed by additional manual curation due to hybrid spectra and potential false positives. Manual curation included additional filtering by the ECN model^45^ (Figure S9) based on the expected elution pattern of our used reversed-phase column. In this way, we identified 137 unique gangliosides structurally resolved at the lipid species or molecular lipid species level in MSC and differentiated samples. In comparison, classical immunological, biochemical, or thin-layer chromatography methods rely on class-specific ganglioside detection. Using our optimized RP-HRMS^n^ ganglioside assay, we obtained both glycan and lipid structural information. To mimick the lower information content provided by a state-of-the-art ganglioside class-specific antibody assay, we summed all detected species (variations in lipid moiety) to obtain a glycan-dependent ganglioside specific quantitative measure for the class level, e.g., GM3, GM4, etc. (Figure 4). We can observe a trend of GD3 upregulation in osteocytes. Considering the concentration on the class level only, we observed GM3 to be highest in adipocytes. A distinct effect of increasing sialic acid length could not be observed, further highlighting that the discriminative power is hidden in the specific ceramide moieties. In contrast to these findings, at the detailed lipid species level, we detected cell type-specific differentiation patterns between all monitored tissue types. Although some trends can be observed at this lowest level of structural resolution, such as (1) higher glycan series in osteocytes or (2) a significantly lower expression of GM2 in MSCs compared to the differentiated cells, important structural information on the ceramide moiety is unavailable. As such, it is not possible to single out the relevant ganglioside species and derive more details about their specific biological functions.

If the ganglioside annotation up to the molecular species level derived by our RP-HRMS^n^ workflow is included, enhanced structural information is obtained compared to traditional ganglioside analysis methods. For example, under each ganglioside class-specific signal, up to 50 individual species could be deconvoluted in our study. The 254 curated gangliosides detected in samples and standards contain ceramide moieties ranging from 32 to 46 carbon atoms and 0 to 2 double bonds. This ganglioside panel included both dihydroxylated and trihydroxylated species with the highest abundance shown by the species GM3 34:1;O2; 34:1:O3, 40:1;O2, 42:1;O2, 42:2;O2, and 42:2;O2 (Table S2). The most prominent LCB was expectedly sphingosine (18:1;O2) in all cell lineages. Most LCBs were dihydroxylated with a length between 16 and 20 carbons, with a predominance for even carbon numbers. If dihydroxylated species were observed, chances of observing the corresponding lower abundant trihydroxylated LCB (e.g., 18:1;O3, 18:2;O3) were higher.

**Figure 5:**
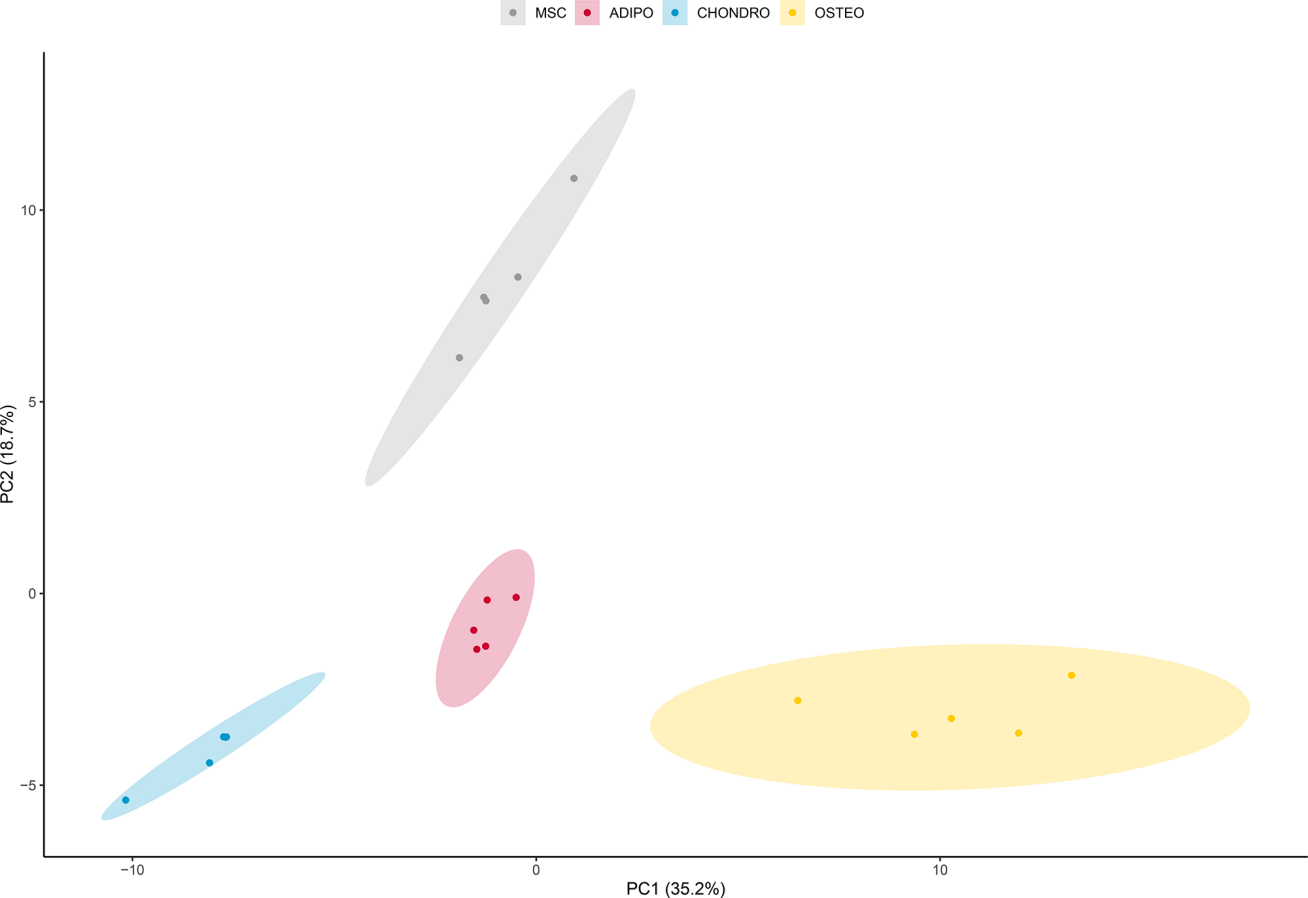
Principal component analysis (PCA) of identified gangliosides species separates MSC and differentiated cell lineages in PC1 and PC2 (number of sample replicates is five). PCA of all 137 quantified unique gangliosides species in biological samples allows separation of cell lineages, with 54% of total variance being explained by PC1 and PC2.

Successful isomer separation was possible by hydrophobic interaction of the lipid part with the reversed-phase column, verified by molecular species assignments using the MS^n^ approach as shown by the example of GM3 18:1;O2/24:1 (retention time: 19.42 min), upregulated in adipocytes, and its isomer GM3 18:2;O2/24:0 (retention time: 19.98 min), upregulated in osteocytes (Figure S3)**.** Interestingly, the total number of ganglioside annotations (unique lipid species per retention time group, “ID_1” in Table S3) increased from mesenchymal stem cells (72) to all differentiated states (adipocytes: 93, chondrocytes 104, osteocytes: 123) (Figure S4). Although we could observe some differences in lipid content of other lipid classes, e.g., TG, PC, and PE were upregulated in adipocytes, compared to the other cell lineages (Figure S5), the overall lipid species content remained almost constant in the bulk lipid classes. In contrast, the ganglioside patterns revealed an increase in variety of ganglioside species upon differentiation and enabled successful cell lineage separation based on distinct ganglioside species (Figure 5).

### Ganglioside Patterns of Native and Differentiated Mesenchymal Stem Cells

In the context of therapeutic applications, it is crucial to maintain the cellular identity and functionalities during *ex vivo* culture to ensure the safety and efficacy of MSCs. However, extensive *ex vivo* expansion of cells is necessary to obtain sufficient cell numbers for therapeutic treatments. Due to frequent passaging on traditional polystyrene surfaces, such as Petri dishes, T-flasks, or well-plates, genetic alterations accumulate over time, resulting in the loss of relevant therapeutic properties.^46−48^ This can impair the therapeutic outcome and pose a safety issue as malignant transformations can be expected.^49^ Therefore, additional markers are desirable to monitor and define the stem cell phenotype during *ex vivo* culture. Several studies including our results indicated gangliosides as promising surface marker candidates for cell lineage, cell state, or function in different biological contexts.^9,20,23,27^ As a proof of concept, we differentiated MSCs toward the adipogenic, chondrogenic, and osteogenic lineage to detect potential ganglioside marker candidates.

**Figure 6:**
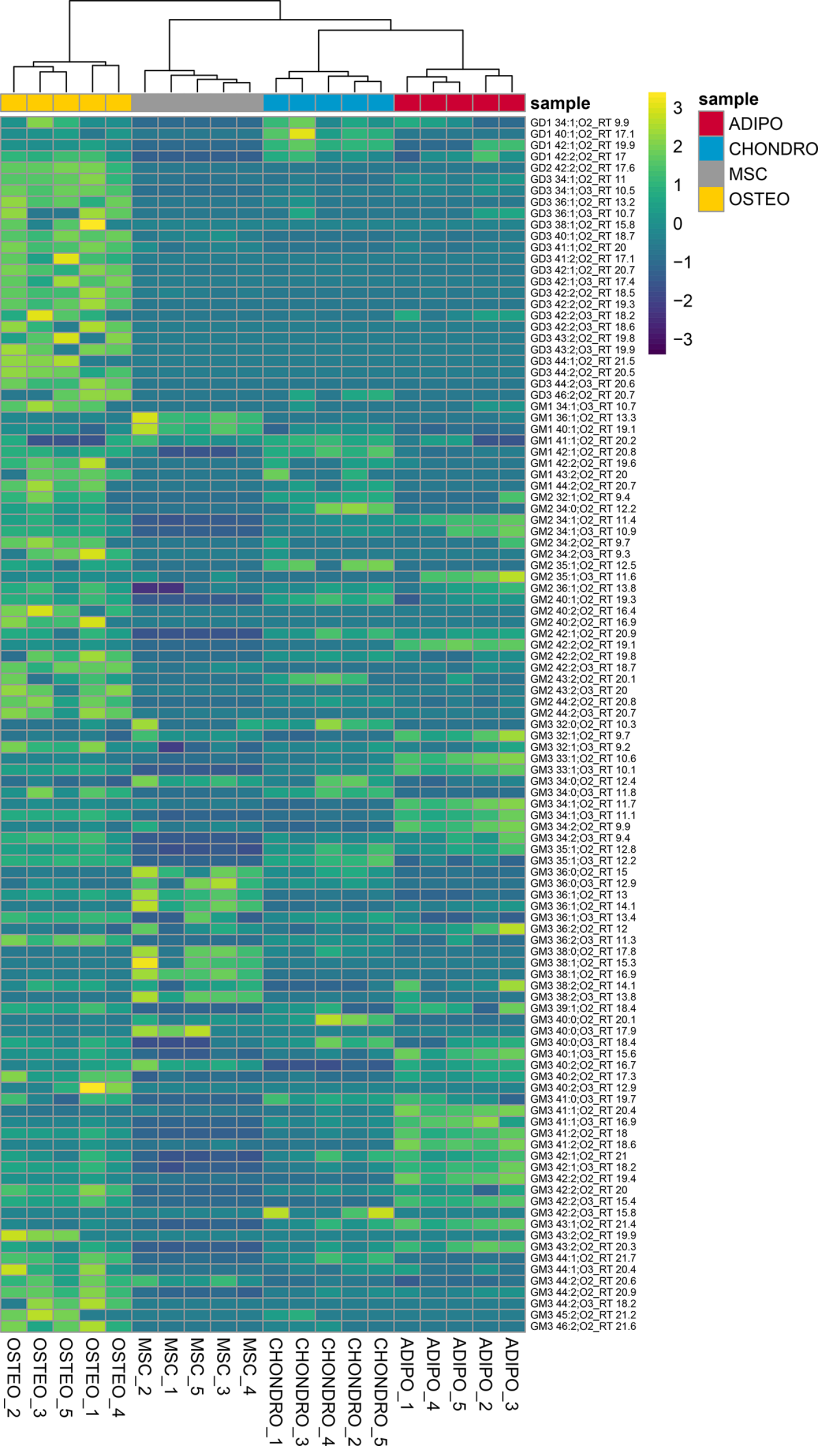
Heatmap of potential marker candidates. Sample replicates (*n* = 5) cluster according to ganglioside regulation patterns observed in negative ionization mode (sum of adducts). Sample names of the different gangliosides include the lipid species and the retention time (in minutes), separated by an underscore. Hierarchical clustering was performed using correlation distance (euclidean) and average linkage scale (ward.D2). The heatmap was plotted using the R package pheatmap.

Out of the 254 identifications with the presented workflow, 137 species from six classes (GM1, GM2, GM3, GD1, GD2, GD3) in native and differentiated MSCs were quantified down to the fmol level, based on one-point calibration with the deuterated internal standard providing concentration estimations. The most abundant gangliosides determined were in the nmol per mg protein range in all samples, where the ganglioside class GM3 showed the highest number of annotations, followed by GD3 and GM2, respectively (Table S1 and Figure S2). For the ganglioside marker selection, we annotated the molecular species level based on MS2 and MS3 fragmentation information wherever possible dependending on ganglioside concentration and quality of spectra (Tables S1 and S3). Significant differences were observed for 78 hits belonging to the classes GD3, GD2, GD1, GM3, GM2, and GM1 (*p*-value < 0.05, Tukey’s HSD, Metaboanalyst (Figure 6)).^50^ By using these 78 candidate markers, the distinction between human MSCs, adipocyte, osteocyte, and chondrocyte sample groups (*n* = 5) was improved (Figure S6) compared to the use of the whole ganglioside pattern (Figure 5).

**Figure 7:**
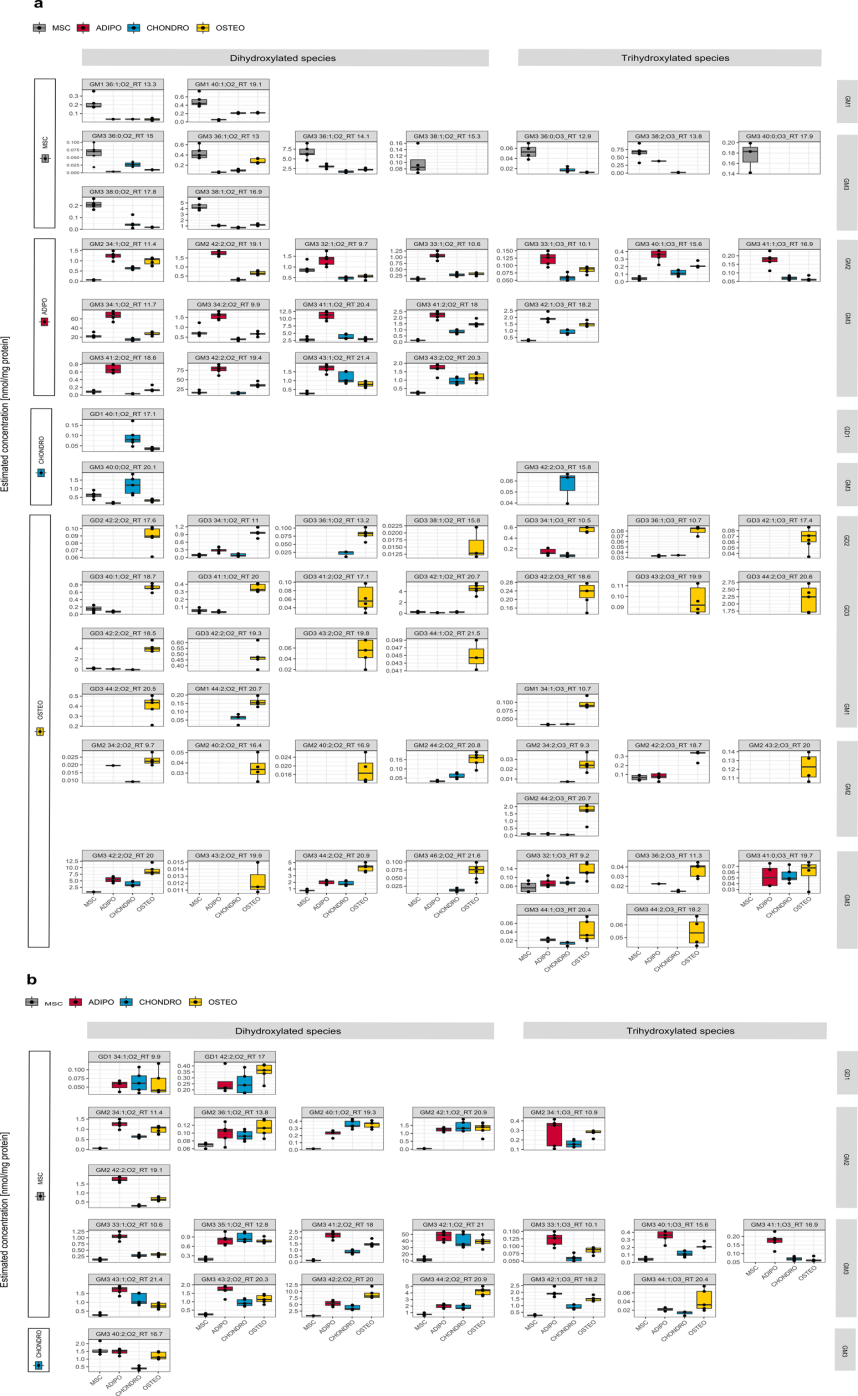
Box plots of significantly regulated potential ganglioside differentiation markers for each cell type. Marker candidates are separated into di- and trihydroxylated ganglioside species and the corresponding ganglioside class. Annotation is based on the generated ID (species level + RT); assigned molecular lipid species levels can be found in Table S3. Estimated concentrations are based on internal standardization with d5 GM1 18:1;O2/18:0 and normalization to the protein content. (a) Upregulated gangliosides for MSC and each differentiated cell lineage. (b) Downregulated gangliosides for MSC and each differentiated cell lineage.

Generally, the classes GM3 and GD3 showed the highest number of potential ganglioside markers. In accordance to our previous data on adipogenesis,^23^ GM3 18:1;O2/16:0 was upregulated only in adipocytes. Despite the observation that the class of GM3 is generally upregulated in adipocytes (Figure 4), some other GM3 species, GM3 36:0;O2, GM3 36:1;O2, GM3 38:0;O2, GM3 38:1;O2, GM3 36:0;O3, GM3 38:2;O3, and GM3 40:0;O3, were also highly upregulated in MSCs, suggesting these lipids as potential non-differentiation/stemness markers (Figure 7). Additionally, two GM1s (GM1 36:1;O2 and GM1 40:1;O2) were also upregulated in MSCs compared to the other cell lines (Figure 7). Conversely, GM2 ganglioside species were generally downregulated or completely absent in MSCs compared to all other differentiated cell lines, indicating GM2 as a differentiation marker (Figures 4 and 7) **+7**). In chondrocytes, we observed the lowest number of potential ganglioside markers (Figure 7). In osteocytes, a general increase of all significantly regulated GD3 gangliosides was observed (Figure 7). A general trend toward longer glycan and ceramide parts in the class of GM3 and GD3 as well as trihydroxylated GM3s is obvious in osteocytes as shown in both the class-specific detection trend in Figure 4 and the marker candidate list depicted in Figure 7.

We detected 78 potential ganglioside markers; from this panel, 38 new gangliosides were reported on the molecular lipid species level (GD3 18:1;O3/16:0, GD3 18:1;O2/26:1, GM3 17:1;O2/16:0, GM3 18:2;O2/16:0, GM3 18:2;O2/18:0, GM3 16:0;O2/22:0, GM3 17:1;O2/22:0, GM3 16:1;O2/24:1, GM3 18:2;O2/22:0, GM3 18:1;O2/23:1, GM3 17:1;O2/24:1, GM3 18:2;O2/24:0, GM3 18:2;O2/24:0, GM3 19:1;O2/24:0, GM3 18:1;O2/25:1, GM3 19:1;O2/24:1, GM3 20:2;O2/23:0, GM3 19:1;O3/23:1, GM3 19:0;O3/23:2) or species level (GD3 41:2;O2, GD3 43:2;O2, GD3 42:2;O3 GD3 42:2;O3, GD3 43:2;O3, GM1 43:2;O2, GM2 43:2;O2, GM2 35:1;O3, GM2 42:2;O3, GM2 43:2;O3, GM2 44:2;O3, GM3 41:2;O2, GM3 43:2;O2, GM3 45:2;O2, GM3 33:1;O3, GM3 35:1;O3, GM3 41:1;O3, GM3 42:2;O3, GM3 44:2;O3) (marked with * in Table S1, detailed ganglioside candidate marker list can be found in Table S3). We defined a lipid species as “novel” if neither present in ChEBI,^51^ HMDB,^52^ LMSD,^3^ SwissLipids,^53^ or the recent comprehensive ganglioside lists published in the literature.^7,25^ The fact that around one third of the high-confidence identifications consisted of novel species proves the general power of the presented RP-HRMS^n^ approach to identify unknown gangliosides in samples of interest with unprecedented structural detail.

## Discussion

Gangliosides have numerous biological roles ranging from their central importance in memory-related processes and diseases to their involvement in cell development and differentiation. State-of-the-art biochemical and immunological analysis methods monitor only the ganglioside class level. As such, they are inadequate to sufficiently resolve the complex ganglioside pattern, which is attributed to both the carbohydrate and lipid moiety, as they are equally important for their biological function.^28^ Native glycolipid analysis is a prerequisite for the evolving field of glyoclipidomics; however, most workflows focus primarily either on the glycan or the lipid moiety. In this work, we established a novel ganglioside assay based on reversed-phase high-resolution mass spectrometry and multistage fragmentation. Highlights of the presented assay are native ganglioside analysis with a sensitivity down to fmol ranges complemented by an automated open-source annotation workflow by LDA, which was recently tailored to sphingolipid structure annotation.^36^ By (1) applying decision rule sets from both MS2 and MS3 spectra deduced from our experimental data in standards and samples, (2) making use of positive and negative ionization mode data, and (3) including retention time information, we significantly improved the ganglioside annotation coverage and quality. Compared to traditional class-specific ganglioside analysis methods,^31^ we were able to deconvolute a single class-specific signal to up to 50 individual ganglioside species, corresponding to an enormous gain in structural information.

As a proof of principle, we applied our novel glycolipidomics assay to characterize the ganglioside profile of native MSCs and MSCs differentiated toward the chondrogenic, adipogenic, and osteogenic lineage. In this study, we reported the highest number of gangliosides so far, encompassing 254 unique ganglioside species which is 1.3–2 times more than in previous reports^7,25,32^ with the additional benefit of automated lipid species annotation offered by our new workflow. The cells in this study fulfilled the minimal criteria for MSCs, including the positive expression of CD75, CD90, and CD105 and the negative expression of CD14, CD20, CD34, and CD45. Furthermore, the cells were successfully differentiated toward the chondrogenic, adipogenic, and osteogenic lineage. The findings of this study are in accordance with observed ganglioside regulations of our previous study on MSCs and adipogenic cell differentiation obtained from a different stem cell donor (female, abdominoplasty).^23^

With our sensitive profiling approach providing increased structural information, we obtained hints to many promising potential biomarker candidates. However, admittedly, some of the detected candidate species may be attributed to donor variability (age, sex, and environmental factors), which affect relevant MSC properties.^54,55^ Furthermore, the cell culture conditions (media and media supplements, culture format, 2D/3D, oxygen level, etc.) may have detrimental effects on all levels of cellular behavior and expression patterns. While these confounding variables do not reduce the analytical power of our assay, to confirm whether our candidates are generally applicable markers for MSC differentiation, additional studies on MSCs from different tissues and donors of different ages and genders are necessary to conclude broad statements on MSC biology. Former studies reported differential expression of GM1, GM3, GD1, and GD2^21,22,56^ in MSCs from different sources. While our results confirm the presence of these classes in undifferentiated MSCs, analysis on the ganglioside class level did not result in a single class that could serve as a sufficient marker for stemness. In this work, we annotated several potential cell-state-dependent ganglioside marker candidates with detailed structural information in MSCs, adipocytes, chondrocytes, and osteocytes (Figure 7). This work indicates that ganglioside profiles may be autonomously used as surrogate markers throughout the whole differentiation states of the three chosen lineages. Although gangliosides can influence membrane shape^57^ and show differentially expressed patterns in different cell types,^19^ little is known to what extent ganglioside patterns affect and/or trigger stemness and stem cell differentiation. Therefore, monitoring the ganglioside profile may be used as a quality control parameter for MSC differentiation in tissue engineering processes and for maintaining stemness during MSC expansion to manufacture cell therapy products. For example, CD markers are defined cell surface molecules that act as targets for immunophenotyping to determine the cell state. Interestingly, the sugar moiety of GD3 serves as a target for quality control and is defined as CD60.^58^ In the context of the presented results, we anticipate to use molecular-level ganglioside-specific markers or marker sets for the characterization of stem cell differentiation processes.

The major novelty presented in this work is the methodological advance using the RP-HRMS^n^ assay for in-depth structural characterization providing both ceramide and glycan information, which are equally important for the ganglioside function.^28^ Current state-of-the-art glycomics workflows focus primarily on the ganglioside class-specific glycan moiety while largely neglecting the lipid moiety. Our approach is complementary in this aspect, as the native gangliosides are analyzed. The multidimensional separation based on chromatography and multistage fragmentation enables both (1) ganglioside identification of classes and isomeric differences as well as (2) quantitative comparison between sample groups. We were further able to identify an extensive ganglioside panel on the lipid species level based on a completely automated annotation by LDA. For ganglioside marker selection in the cell samples, the molecular species level based on MS2 and MS3 fragmentation information was assigned by manual curation wherever possible (depending on ganglioside concentration and quality of spectra). We identified 38 gangliosides never reported so far, proving the general power of the presented RP-HRMS^n^ approach to identify novel ganglioside molecular species at a highly structurally resolved level. However, while we now obtain both the glycan and the lipid information at the same time up to the (molecular) lipid species level, specific glycan isomers such as positional sialic acid isomers (a/b series) or sugar branching isomers (e.g., GM1 and sialysated lacto series) cannot be distinguished by solely analyzing fragmentation spectra so far (more information pertaining to ganglioside structure see Figure S1). While such differentiation would be highly desirable to avoid false positive annotation, the lack of isomeric pairs of authentic standards for those classes prevented us from further pursuing the development of decision rules to optimize the LDA toward unambiguously distinguishing them. Consequently, in this work, we based ganglioside annotation on aligning the reversed-phase retention times of the identifications to the ones of commercially available ganglioside standards. Moreover, the LDA predicts the retention times of other species based on the detections in a sample.^37^ Since upon manual inspection the predicted retention times coincided with the actual hits and there are no isomeric peaks present at other retention times, we regarded this issue of minor importance, at least in our samples. However, by applying our method to other samples, one has to be aware that such potential glycan isomeric overlaps may arise. Thus, we recommend to perform a retention time plausibility check by aligning the retention times to reference standards measured in a separate run. In the future, we anticipate that additional chromatographic separation and improved MS-instrumentation providing complementary fragmentation information exploitable by LDA decision rules will resolve this issue and remove the requirement for this additional plausibility check. For this purpose, we will need iso-pure individual ganglioside and glycolipid standards, which are currently commercially unavailable. Our presented open-source annotation workflow based on LDA can be easily applied to any other hyphenated liquid chromatography and MS setup by including new and/or adapting existing decision rules reflecting fragmentation information obtained by (ideally iso-pure) glycolipid standards. We already demonstrated that different glycolipid classes can be added using the presented glycolipidomics annotation, e.g., for glycosyl inositol phospho ceramides in plants.^59^ Hence, our LDA-based annotation strategy is currently the only automated glycolipid annotation software available for the community.

Overall, our proposed workflow can be seen as the hallmark for shedding light on the enigmatic processes of gangliosides and glycolipids in general, which are critical mediators in cell–cell interaction and signaling pathways. Hence, we believe multidimensional separation and HRMS^n^-based assays will lay the foundation to unravel how glycolipids orchestrate the fatty sweet symphony in cells and organisms. Moreover, our workflow will be an innovative strategy to determine and make use of novel glycolipid-based CD markers for stem cell phenotype characterization.

## Methods

### Cell Culture

The use of human tissue was approved by the ethics committee of the University of Lübeck (EK Nr: 20-333), and the donor (male, 29 years, adipose tissue from abdominoplasty) provided written consent. MSCs were isolated within approximately 12 h after surgery as previously described.^60^ MSCs were cultured in a standard medium composed of MEM alpha (Thermo Fisher Scientific, Waltham, MA, USA), 0.5% gentamycin (Lonza, Basel, Switzerland), 2.5% human platelet lysate, and 1 IU/mL heparin (both PL BioScience, Aachen, Germany) on standard T-flasks (Sarstedt, Nümbrecht, Germany), in a humidified incubator at 37 °C and 5% CO_2_. After confluency was reached, cells were detached with accutase (Sigma Aldrich) and cryo-preserved in liquid nitrogen as previously described.^61^ Upon use, MSCs were thawed and subcultivated in T-flasks once, resulting in passage two.

### Differentiation

For MSC differentiation, 4000 cells/cm^2^ in passage two were seeded on a 6-well plate (Sarstedt) coated with fibronectin (2 μg/cm^2^; Sigma Aldrich) and allowed to grow confluent. The medium was changed to adipogenic, chondrogenic, or osteogenic medium (Miltenyi Biotech, Bergisch Gladbach, Germany, supplemented with 0.5% gentamycin, *n* = 5 replicates for each medium). Cells were cultivated for 21 days, and the medium was changed every 3 to 4 days. The confluent MSCs at day zero of differentiation served as control.

### Histological Staining

MSCs from adipogenic and chondrogenic mediums were fixed with 4% paraformaldehyde (PFA, both Sigma). Cells cultured in adipogenic differentiation medium were stained with Oil Red O (Sigma Aldrich) for lipid vacuoles. Cells were stained for glycosaminoglycans with Alcian blue (Sigma Aldrich) to confirm chondrogenic differentiation. MSCs cultivated in the osteogenic medium were fixed with 96% ethanol and stained for calcium with Calcein and nuclei counterstained with DAPI (Sigma). Cells at day zero served as control.

### Phenotyping

To determine MSC surface marker expression, MSCs in passage two were detached by accutase treatment and stained with a human MSC phenotyping kit and an anti-HLA-DR antibody (both Miltenyi Biotec) according to the manufacturer’s instructions. In this kit, the antibodies for the negative markers (CD14, CD20, CD34, and CD45) are labeled with the same fluorophore to generate a negative marker panel. According to the manufacturer’s manual, an approximate 10-fold increase of the fluorescence intensity of the negative markers is expected for negative samples compared to the isotype control. The stained cells were resuspended in a suitable volume of flow cytometry buffer (0.5% fetal bovine serum, two mM EDTA in PBS), and 1 × 10^4^ gated events per sample were recorded on a CytoFLEX S (Beckman Coulter, Brea, CA, USA). Subsequent analysis was performed with Kaluza Flow Cytometry software (version 1.3, Beckman Coulter).

### Glycolipidomics

Detailed information on the glycolipidomics workflow can be found in the extended methods section in the Supporting Information.

#### Standards and Solvents

All solvents were LC–MS grade. Ganglioside standards were from Cayman Chemical (GD2, GM4, Ann Abor, USA) or Avanti Polar Lipids, Inc. (GM3 bovine milk, total ganglioside extract from porcine brain, Alabaster, Alabama, USA) and were weighed and dissolved in an appropriate solvent (IPA/H_2_O (65%/35%, v/v)). Deuterated d5 GM1 18:1;O2/18:0 standard was purchased from Avanti Polar Lipids, Inc. (Alabaster, Alabama, USA) and used as an internal standard for further analysis.

#### Ganglioside Extraction

An adapted SIMPLEX protocol^62^ was used to extract gangliosides as described previously^23^ since it enables the simultaneous collection of lipids (upper phase), metabolites (lower phase), and the protein pellet. In short, MSC and differentiated cell lineages (adipocytes, chondrocytes, and osteocytes) were quenched directly on the 6-well plate before storage at −80 °C until sample preparation. Deuterated internal standard was added directly to the samples to compensate for losses during extraction. Harvesting of adherent cells (∼2 × 10^5^ cells/well) was performed using a cell scraper. Subsequent extraction was accomplished using a mixture of cold methanol, methyl-*tert*-butyl ether (MTBE), and 10 mM ammonium formate. Five replicates/conditions, as well as four medium blanks (control), were prepared. The lipid fractions were collected, dried, and reconstituted in IPA/H_2_O (65%/35%, v/v). To determine the protein concentration, the BCA assay (Pierce kit, Thermo Fisher) was applied.

#### Ganglioside Profiling with RP-HRMS^n^

A tailored ganglioside method was developed using a Vanquish Horizon UHPLC system coupled via heated electrospray ionization (HESI) to a high-field Orbitrap ID-X Tribrid mass spectrometer (both from Thermo Fisher Scientific). RP chromatography was accomplished using an Acquity HSS T3 (2.1 mm × 150 mm, 1.8 μm, Waters) column with a VanGuard pre-column (2.1 mm × 5 mm, 100 Å, 1.8 μm). The column temperature was set to 40 °C, the flow rate was set to 0.25 mL min^–1^, and the injection volume was set to 5 μL. The injector needle was flushed with 75% isopropanol (IPA) and 1% formic acid in between the injections. Acetonitrile (ACN)/H2O (3:2, v/v) was used as solvent A and IPA/ACN (9:1, v/v) as solvent B, both containing 0.1% formic acid and 10 mM ammonium formate. A gradient of 30 min under following conditions was applied: 0.0–2.0 min 30% B, 2.0–3.0 min ramp to 55% B, 3.0–17.0 min ramp to 67% B, 17.0–22.0 min ramp to 100%, 22.0–26.0 min 100% B, 26.0 min fast switch to 30% B, and 26.0–30.0 equilibration at starting conditions (30% B).

The ESI source parameters were as follows: 3.5 (positive ion mode) and 3.0 kV (negative ion mode), sheath gas 40, auxiliary gas 8, sweep gas 1, capillary temperature (ion transfer tube temperature) 275 °C, auxiliary gas heater (vaporizer temperature) 350 °C, radio frequency (RF) level 45%. Positive and negative ionization mode data were acquired in separate runs.

Spectral data were acquired in profile mode. For full MS runs, a mass range of *m*/*z* 500–2000 at a resolution of 120,000 was selected. The automatic gain control (AGC) target was set to standard; the maximum injection time (MIT) was 100 ms. MS2 and MS3 scans were performed with data-dependent acquisition (DDA). For MS2 scanning, a top 5 method with a resolution of 15,000 was applied. The normalized collision energy (NCE) was set to 23 (+) and 27 (−) (HCD activation), the isolation window was set to *m*/*z* 1.5, the AGC target was set to standard, and the MIT was set to 60 ms. The dynamic exclusion of triggered *m*/*z* was set to 5 s, and a ganglioside-specific inclusion list was implemented. For MS3 spectra, the mass range was reduced to *m*/*z* 300–800 to gain fragments of the ceramide moieties (LCB, FA) to elucidate the molecular lipid species composition. The Ion Trap was selected as a mass analyzer with a fixed collision energy of 30% (CID activation), with 10 ms activation time and an activation Q of 0.25. The Ion Trap scan rate was set to rapid, and the isolation windows were set to *m*/*z* 1.5 (MS1) and 2.0 (MS2), respectively, the AGC target was set to standard and MIT to automatic. The MS3 scans in the Ion trap were parallelizable with MS1 and MS2 scans in the Orbitrap, increasing information content and saving time (Figure 2).

Deep ganglioside and lipid profiling was performed on sequential automated exclusion lists (including blank subtraction) enabled by AcquireX data acquisition software within the Orbitrap ID-X Tribrid mass spectrometer. More information on the RP-HRMS^n^-based ganglioside profiling can be found in the extended method section of the Supporting Information.

### Data Evaluation

The ganglioside assignment was performed using LDA.^36,63^ The applied *OrbiTrap_IDX_heavy* settings as well as decision rule sets and corresponding mass lists are included starting with LDA version 2.8.3, which is available for download at LDA homepage http://genome.tugraz.at/lda2. All analysis results in both polarities were processed together (Statistical Analysis), as it provides retention time alignment (RT group). The results were subsequently exported using the rdb export option. All adduct areas of the internal standard were received from Skyline (version 21.2) and exported as a csv file. LDA (ganglioside annotations), Skyline (internal standard), and BCA (protein content) results were then evaluated using R Studio (version 4.2.1). Unique ganglioside hits (IDs) were curated using the lipid species as well as the retention time group information of the LDA export (see “ID_1” in Table S3). Several quality control filters were applied, including (1) retention time filter (2–26 min), (2) ppm filter (max. 5 ppm), (3) area threshold filter (min 30,000), (4) single annotation filter (at least three detections over all files), and (5) MS2 filter (at least one MS2 spectra in standard or sample file). Moreover, only trihydroxylated species with a corresponding dihydroxylated species were considered. Retention time matching of adducts in both polarities were performed on the generated ID. The sum of adducts was calculated for each polarity and each ID. All annotations were normalized to the sum of adduct of the internal standard (IS d5 GM1 36:1;O2) in both polarities. The lipid species’ concentration was estimated via one-point calibration using the known concentration of the internal standard. Subsequently, normalization to the protein content was performed, followed by calculation of mean and standard deviation for sample replicates. As a final filter, the ECN models for each ganglioside class were plotted and manually filtered, whereby fitting retention times according to the ECN model, MS1 peak shape, and MS2 spectral quality were considered.

Data was exported to MetaboAnalyst^50^ (version 5.0) for visualization and statistical analysis (one-factor). Results were exported and plotted using R Studio.

Positive ion mode data was predominantly used to corroborate the annotations detected in negative ion mode (matching RTs) and where possible, for obtaining annotations at the molecular lipid species level. Quantities presented in figures and tables are the sum of adduct abundances detected in negative ion mode, as this ion mode provided better coverage with respect to the number of detected species. Detailed information on the data evaluation workflow can be found in the extened method section in the Supporting Information.

## Supporting Information Available

Ganglioside class and annotation overview, overview of identified gangliosides in native and differentiated MSCs, benefit of combining reversed-phase chromatography and multistage fragmentation for ganglioside annotation, number of gangliosides species identified in different cell states, lipid profiling of stem, fat, bone, and cartilage cell pools, PCA of potential marker candidates, list of significantly regulated ganglioside species and manually assigned molecular lipid species , extended methods, protein content, MS^n^ workflow, fragmentation rule example, Skyline for internal standards, ECN model at the example of identified GM2 gangliosides, applied filters during data processing, and effect of applied filters to different sample types (PDF)
Overview of gangliosides marker (XLSX)
Annotated gangliosides in all data (XLSX)

## Notes

All data are included in the article and the Supporting Information or are available from the authors upon request. The open-access version of LDA for ganglioside analysis is freely available at http://genome.tugraz.at/lda2, as well as the (1) raw data, (2) mzml files, and (3) general ganglioside annotation list at GNPS (WinSCP client has to be downloaded, ftp://MSV000089064@massive.ucsd.edu).

## Notes

The isolation of MSCs from human adipose tissue was approved by the ethics committee of the University of Lübeck (EK Nr: 20-333), and the donor (male, 29 years) provided written consent.
