# Supplementary material for: Decoding Distinct Ganglioside Patterns of Native and Differentiated Mesenchymal Stem Cells by a Novel Glycolipidomics Profiling Strategy
Source: JACS Au. 2022 Oct 25;2(11):2466–80. doi: 10.1021/jacsau.2c00230 (PMC9709940; doi:10.1021/jacsau.2c00230)
Supplement: Supplementary file 1 — au2c00230_si_001.pdf [file au2c00230_si_001.pdf]

Supporting information

Decoding Distinct Ganglioside Gatterns of Native and Differentiated Mesenchymal Stem Cells by a Novel Glycolipidomics Profiling Strategy

Katharina Hohenwallner[1,2], Nina Troppmair[1,2], Lisa Panzenboeck[1,2], Cornelia Kasper[3], Yasin El Abiead[1], Gunda Koellensperger[1], Leonida M. Lamp[4], Jürgen Hartler[4,5], Dominik Egger[3], Evelyn Rampler*[1]

[1] Department of Analytical Chemistry, Faculty of Chemistry, University of Vienna, 1090, Vienna, Austria

[2] Vienna Doctoral School in Chemistry (DoSChem), University of Vienna, 1090, Vienna, Austria

[3] Institute of Cell and Tissue Culture Technologies, University of Natural Resources and Life Sciences, 1190, Vienna, Austria

[4] Institute of Pharmaceutical Sciences, University of Graz, 8010, Graz, Austria

[5] Field of Excellence BioHealth, University of Graz, 8010, Graz, Austria

Supporting information
This section contains the supplementary (S) figures and tables

Supporting information

Figure S1: Ganglioside class and annotation overview........ 3

Figure S2: Overview of identified gangliosides in native and differentiated MSCs........ 4

Figure S3: The benefit of combining reversed-phase chromatography and multistage fragmentation for ganglioside annotation........ 5

Figure S4: Number of gangliosides species identified in different cell states........ 6

Figure S5: Lipid profiling of stem, fat, bone, and cartilage cell pools........ 7

Figure S6: PCA of potential marker candidates........ 7

Table S1: Overview of ganglioside markers........ 8

Table S2: Annotated gangliosides in all data........ 8

Table S3: List of significantly regulated ganglioside species and manually assigned molecular lipid species level........ 9

Extended Methods........ 12

Standards and solvents........ 12

Ganglioside extraction........ 12

Table S4: Protein content........ 12

Ganglioside profiling with RP-HRMS^n........ 13

Figure S7: MS workflow........ 14

Data evaluation........ 14

Table S5: Fragmentation rule example........ 15

Figure S8: Skyline for internal standards........ 17

Figure S9: ECN model at the example of identified GM2 gangliosides........ 18

Table S6: Applied filters during data processing........ 19

Figure S10: Effect of applied filters to different sample types........ 20

Figure S1: Ganglioside class and annotation overview. a Structures of 12 different ganglioside classes containing one (GM1-4), two (GD1-3), three (GT1-3), four (PQ1) or five (GP1) sialic acid residues. In accordance with the LIPID MAPS Structural Database definition of gangliosides, we consider just ganglioside series containing at least one sialic acid[1]. The sialic acid positional isomers for ganglioside glycan subclasses (e.g. GM1 a/b) cannot be distinguished using the applied RP-HRMS^n workflow. **b** Ganglioside annotation based on the lipid class, species or molecular lipid species level. **c** Core sequences of neutral sugars according to LipidWeb[2]. Hexoses (glucose, galactose, mannose) cannot be differentiated by MS/MS spectra: Isomeric glycan sequences with identical lipid parts such as sialylated lacto-, neolacto-, and globo-series cannot be distinguished from the respective ganglioside using the presented RP-HRMS^n workflows (e.g. GM1, LM1), due to the lack of fragments distinct for the respective hexose moiety.

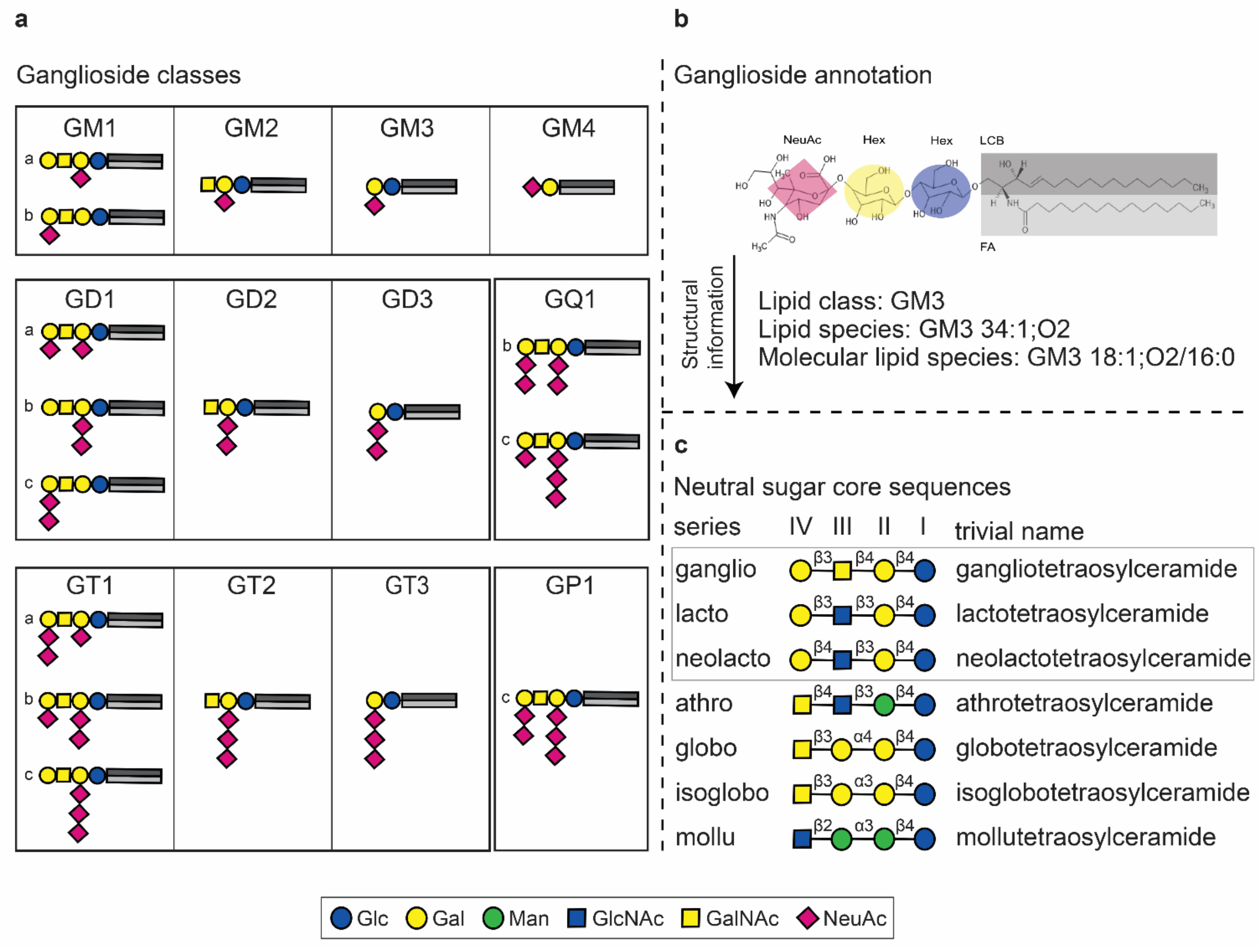

Figure S2: Overview of identified gangliosides in native and differentiated MSCs. 110 unique gangliosides were identified on the lipid species level (sum of adducts, negative ion mode) including estimated concentration based on internal standardization with d5 GM1 18:1;O2/18:0 and normalization to the protein content.

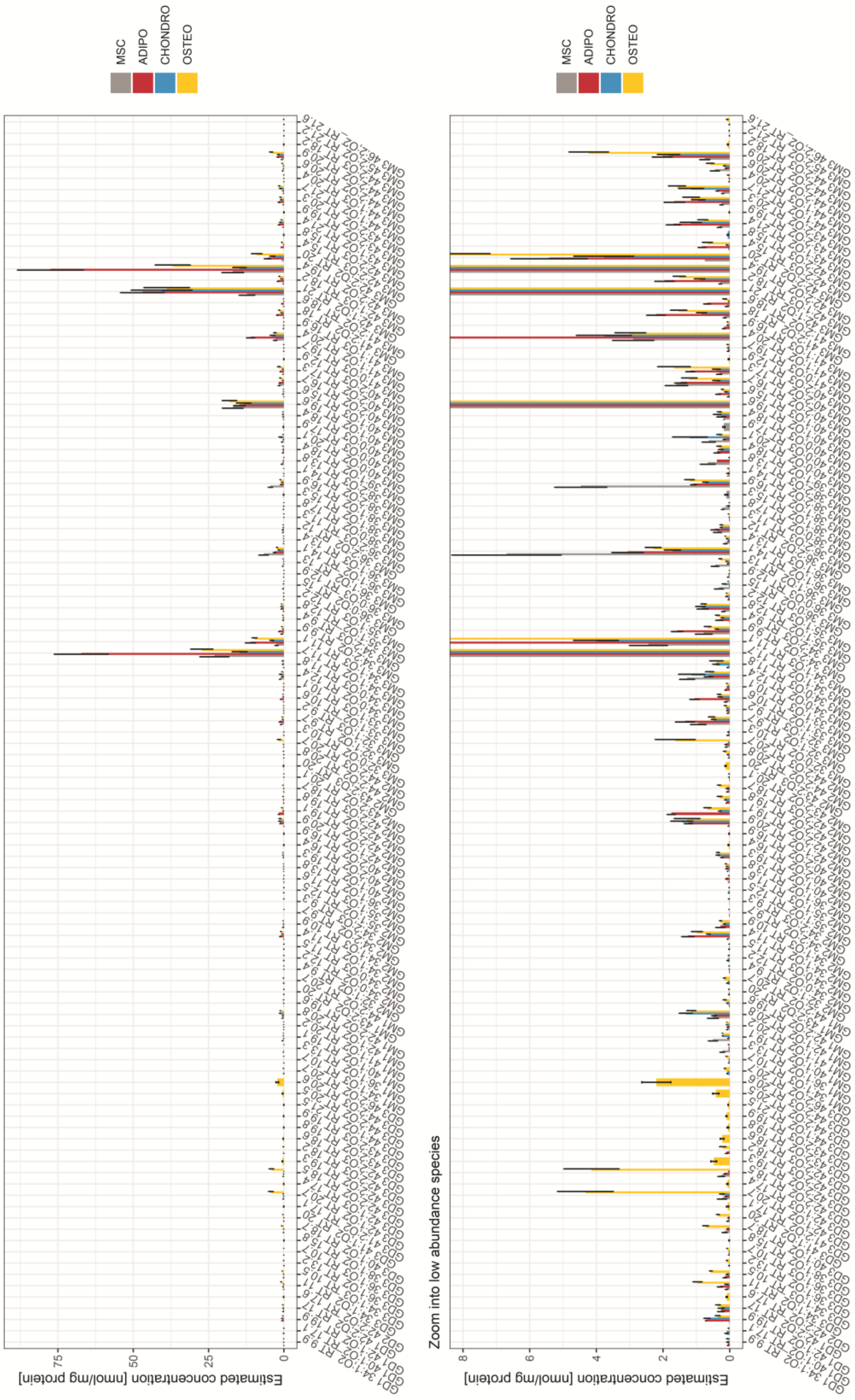

Figure S3: The benefit of combining reversed-phase chromatography and multistage fragmentation for ganglioside annotation. RP-HRMS^n enables isomeric separation of GM3 18:1;O2/24:1 at a retention time 19.40 min and GM3 18:2;O2/24:0 at retention time of 19.96 min followed by molecular species level assignment based on the fragmentation of the glycan and ceramide part in MS2 and MS3 (positive ion mode). GM3 18:1;O2/24:1 is an isomeric marker for adipocytes compared to GM3 18:2;O2/24:0 being upregulated in osteocytes.

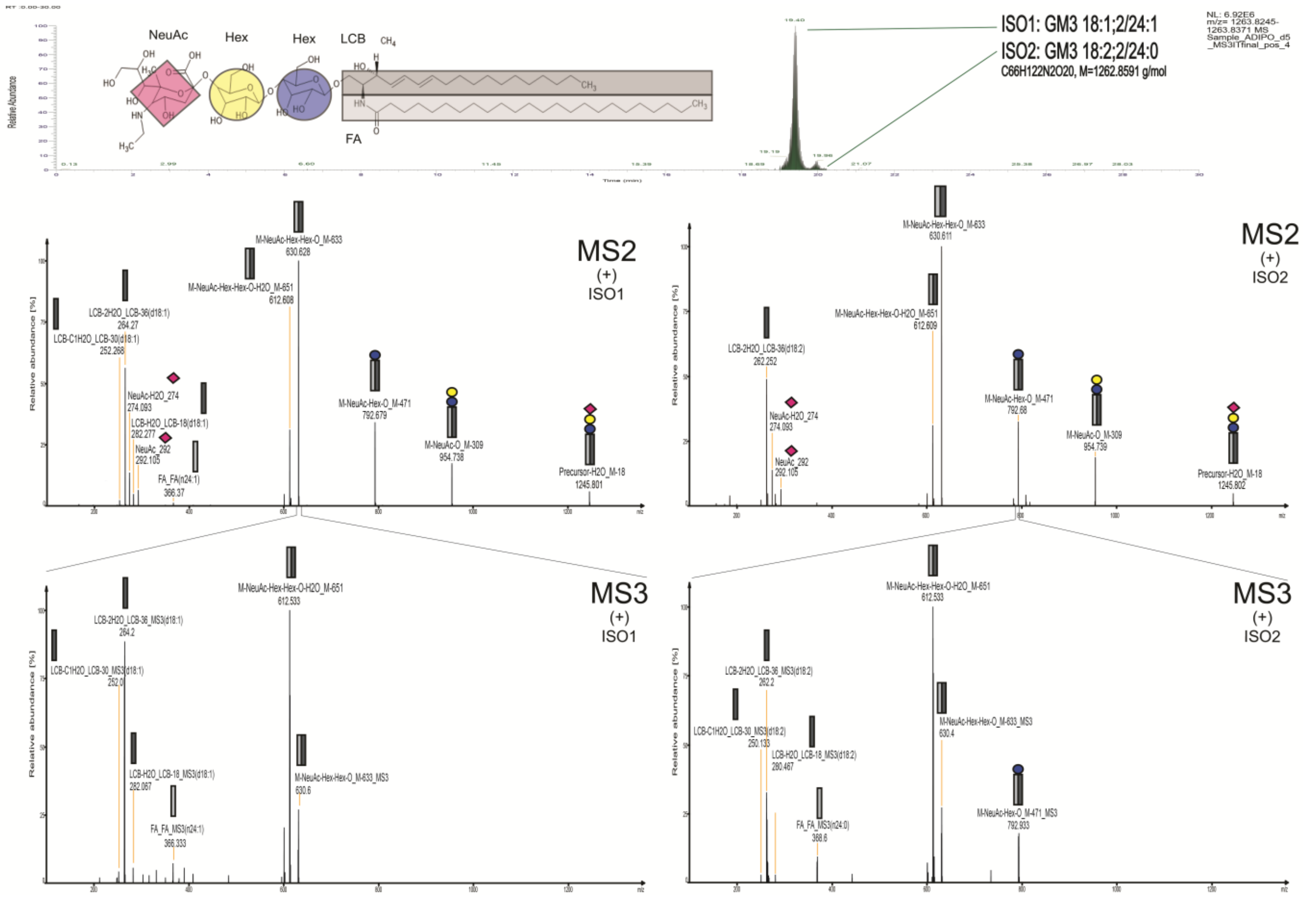

The annotation shown in MS2 and MS3 spectral data is based on user-specific nomenclature within the applied fragmentation rules of the LDA software. Moreover, visualization for glycan and lipid have been applied.

Figure S4: Number of gangliosides species identified in different cell states. An increasing number of ganglioside species was observed in all differentiated cells states (fat, bone, cartilage), with the highest number of gangliosides identified in osteocytes for both positive and negative ionization. Numbers were counted by unique ID (lipid species per RT group) for each sample group and polarity.

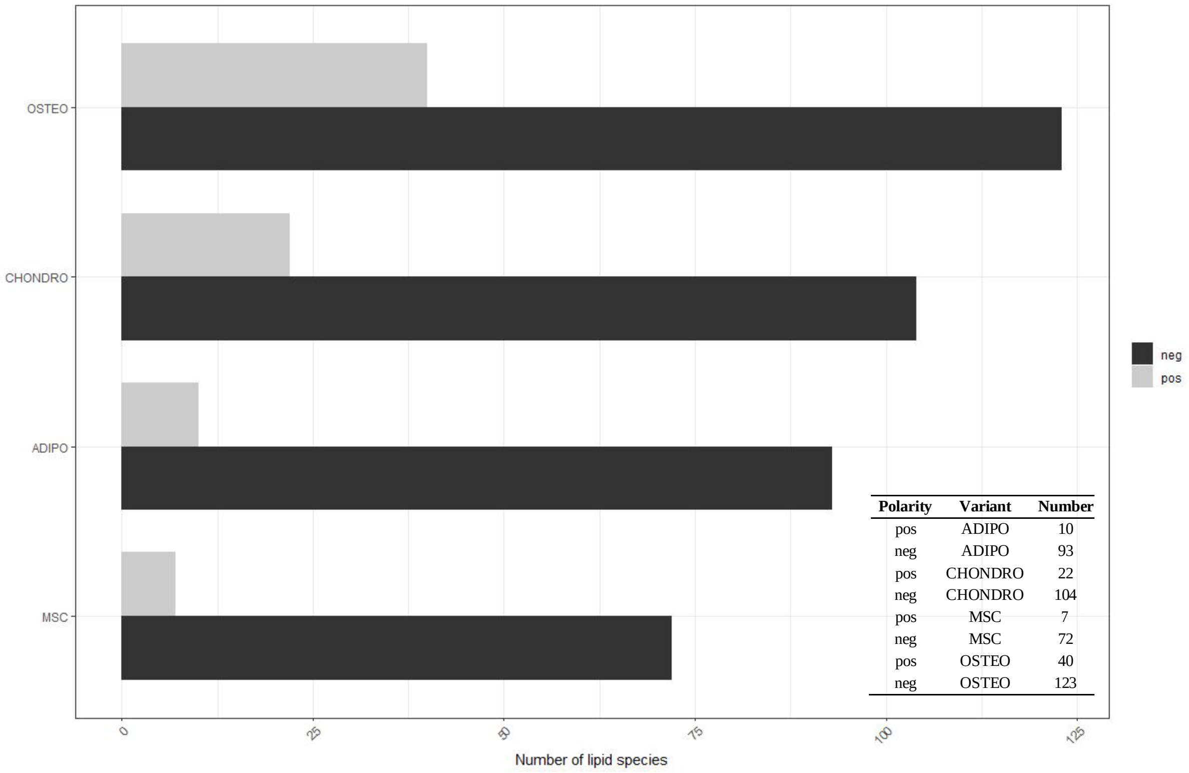

Polarity	Variant	Number
pos	ADIPO	10
neg	ADIPO	93
pos	CHONDRO	22
neg	CHONDRO	104
pos	MSC	7
neg	MSC	72
pos	OSTEO	40
neg	OSTEO	123

Figure S5: Lipid profiling of stem, fat, bone, and cartilage cell pools. For fat cells, TG, DG and PC are present in higher abundance. A potential cause may be the role of adipocytes acting as storage organelles for neutral lipids. Additionally, higher amounts of ceramides are detectable in cartilage (chondrocyte) cells.

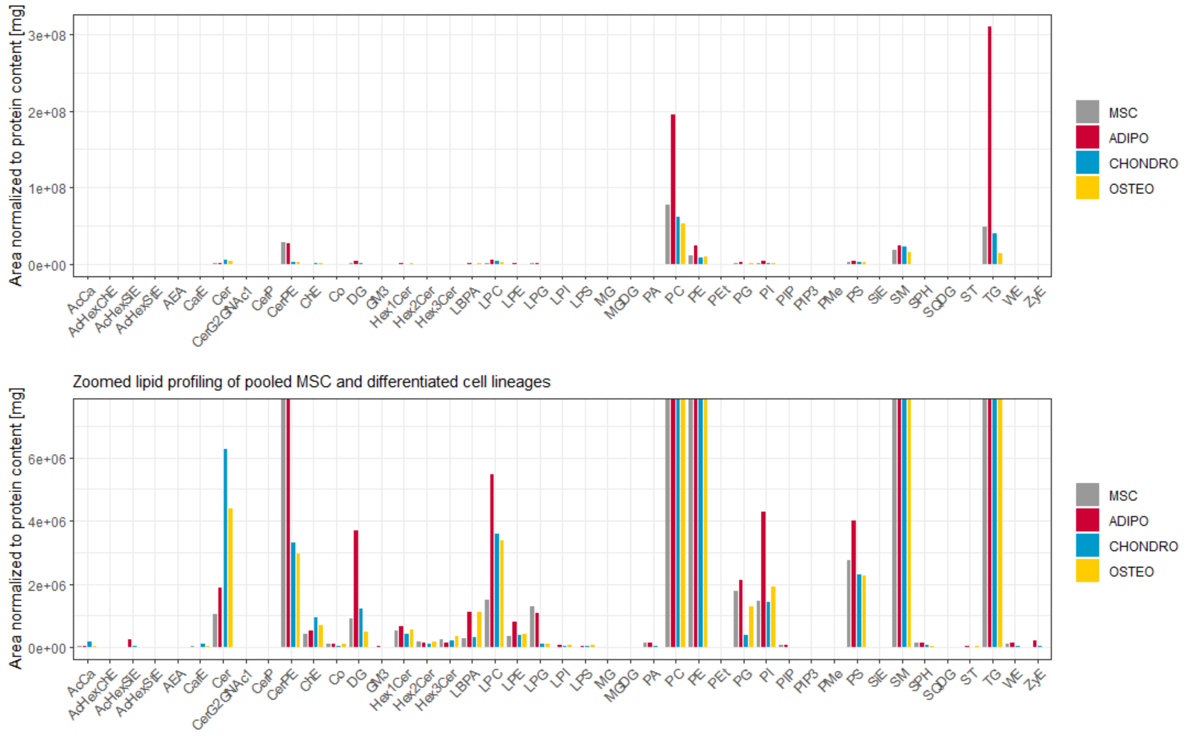

Figure S6: PCA of potential marker candidates. Principal component analysis (PCA) of 78 significantly regulated (p-value < 0.05) gangliosides species separates MSC and differentiated cell lineages in PC1 and PC2 (number of sample repeats is five). 63% of total variance were explained by PC1 and PC2.

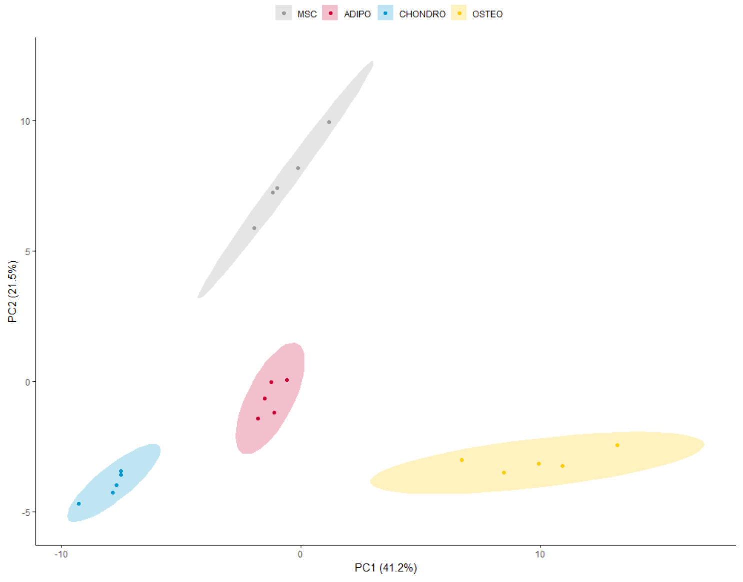

Table S1: Overview of ganglioside markers. List of annotated gangliosides (sum of adducts) in native and differentiated MSCs (n=5), including estimated concentrations based on normalization to internal standard d5 GM1 d36:1;O2 and the protein content. Previously unreported novel ganglioside molecular species are indicated by *. Species level annotation is provided in both old (GM3 d36:1) and new (GM3 36:1;O2) nomenclature to avoid misinterpretation. Estimated concentrations are based on normalization to IS d5 GM1 d36:1;O2 and the protein content.

Table S2: Annotated gangliosides in all data. List of all 254 identified gangliosides (all adducts) in native and differentiated MSCs (n=5), as well as external standards, blanks and QC files. Information of detected fragments in MS2 and MS3 spectra can be found for every file.

Table S3: List of significantly regulated ganglioside species and manually assigned molecular lipid species level. 38 ganglioside (molecular) lipid species were reported for the first time and are marked with *. In some cases the molecular lipid species assignment revealed hybrid spectra of coeluting isomeric species (marked in dark grey).

NO.	CLASS	SPECIES	SPECIES NEW	MOLSPECLEVEL	ID_1	RT [min]
**1**	GD1	GD1 d34:1	GD1 34:1;O2		GD1 d34:1_9.9	9.9
**2**	GD1	GD1 d40:1	GD1 40:1;O2		GD1 d40:1_17.1	17.1
**3**	GD1	GD1 d42:1	GD1 42:1;O2		GD1 d42:1_19.9	19.9
**4**	GD1	GD1 d42:2	GD1 42:2;O2		GD1 d42:2_17	17.0
**5**	GD2	GD2 d42:2	GD2 42:2;O2		GD2 d42:2_17.6	17.6
**6**	GD3	GD3 d34:1	GD3 34:1;O2	GD3 18:1;O2/16:0	GD3 d34:1_11	11.0
**7**	GD3	GD3 d36:1	GD3 36:1;O2	GD3 18:1;O2/18:0	GD3 d36:1_13.2	13.2
**8**	GD3	GD3 d38:1	GD3 38:1;O2	GD3 18:1;O2/20:0	GD3 d38:1_15.8	15.8
**9**	GD3	GD3 d40:1	GD3 40:1;O2		GD3 d40:1_18.7	18.7
**10**	GD3	GD3 d41:1	GD3 41:1;O2		GD3 d41:1_20	20.0
**11**	GD3	GD3 d41:2	GD3 41:2;O2*		GD3 d41:2_17.1	17.1
**12**	GD3	GD3 d42:1	GD3 42:1;O2	GD3 18:1;O2/24:0	GD3 d42:1_20.7	20.7
**13**	GD3	GD3 d42:2	GD3 42:2;O2	GD3 18:1;O2/24:1	GD3 d42:2_18.5	18.5
**14**	GD3	GD3 d42:2	GD3 42:2;O2		GD3 d42:2_19.3	19.3
**15**	GD3	GD3 d43:2	GD3 43:2;O2*		GD3 d43:2_19.8	19.8
**16**	GD3	GD3 d44:1	GD3 44:1;O2		GD3 d44:1_21.5	21.5
**17**	GD3	GD3 d44:2	GD3 44:2;O2		GD3 d44:2_20.5	20.5
**18**	GD3	GD3 d46:2	GD3 46:2;O2		GD3 d46:2_20.7	20.7
**19**	GD3	GD3 t34:1	GD3 34:1;O3	GD3 18:1;O3/16:0*	GD3 t34:1_10.5	10.5
**20**	GD3	GD3 t36:1	GD3 36:1;O3		GD3 t36:1_10.7	10.7
**21**	GD3	GD3 t42:1	GD3 42:1;O3		GD3 t42:1_17.4	17.4
**22**	GD3	GD3 t42:2	GD3 42:2;O3*		GD3 t42:2_18.2	18.2
**23**	GD3	GD3 t42:2	GD3 42:2;O3*		GD3 t42:2_18.6	18.6
**24**	GD3	GD3 t43:2	GD3 43:2;O3*		GD3 t43:2_19.9	19.9
**25**	GD3	GD3 t44:2	GD3 44:2;O3	GD3 18:1;O2/26:1*	GD3 t44:2_20.6	20.6
**26**	GM1	GM1 d36:1	GM1 36:1;O2	GM1 18:1;O2/18:0	GM1 d36:1_13.3	13.3
**27**	GM1	GM1 d40:1	GM1 40:1;O2		GM1 d40:1_19.1	19.1
**28**	GM1	GM1 d41:1	GM1 41:1;O2		GM1 d41:1_20.2	20.2
**29**	GM1	GM1 d42:1	GM1 42:1;O2		GM1 d42:1_20.8	20.8
**30**	GM1	GM1 d42:2	GM1 42:2;O2		GM1 d42:2_19.6	19.6
**31**	GM1	GM1 d43:2	GM1 43:2;O2*		GM1 d43:2_20	20.0
**32**	GM1	GM1 d44:2	GM1 44:2;O2		GM1 d44:2_20.7	20.7
**33**	GM1	GM1 t34:1	GM1 34:1;O3		GM1 t34:1_10.7	10.7
**34**	GM2	GM2 d32:1	GM2 32:1;O2		GM2 d32:1_9.4	9.4
**35**	GM2	GM2 d34:0	GM2 34:0;O2		GM2 d34:0_12.2	12.2
**36**	GM2	GM2 d34:1	GM2 34:1;O2	GM2 18:1;O2/16:0	GM2 d34:1_11.4	11.4
**37**	GM2	GM2 d34:2	GM2 34:2;O2		GM2 d34:2_9.7	9.7
**38**	GM2	GM2 d35:1	GM2 35:1;O2		GM2 d35:1_12.5	12.5
**39**	GM2	GM2 d36:1	GM2 36:1;O2		GM2 d36:1_13.8	13.8

40	GM2	GM2 d40:1	GM2 40:1;O2		GM2 d40:1_19.3	19.3
41	GM2	GM2 d40:2	GM2 40:2;O2		GM2 d40:2_16.4	16.4
42	GM2	GM2 d40:2	GM2 40:2;O2		GM2 d40:2_16.9	16.9
43	GM2	GM2 d42:1	GM2 42:1;O2		GM2 d42:1_20.9	20.9
44	GM2	GM2 d42:2	GM2 42:2;O2		GM2 d42:2_19.1	19.1
45	GM2	GM2 d42:2	GM2 42:2;O2		GM2 d42:2_19.8	19.8
46	GM2	GM2 d43:2	GM2 43:2;O2*		GM2 d43:2_20.1	20.1
47	GM2	GM2 d44:2	GM2 44:2;O2		GM2 d44:2_20.8	20.8
48	GM2	GM2 t34:0	GM2 34:0;O3		GM2 t34:0_11.5	11.5
49	GM2	GM2 t34:1	GM2 34:1;O3	GM2 18:1;O3/16:0	GM2 t34:1_10.9	10.9
50	GM2	GM2 t34:2	GM2 34:2;O3		GM2 t34:2_9.3	9.3
51	GM2	GM2 t35:1	GM2 35:1;O3*		GM2 t35:1_11.6	11.6
52	GM2	GM2 t42:2	GM2 42:2;O3*		GM2 t42:2_18.7	18.7
53	GM2	GM2 t43:2	GM2 43:2;O3*		GM2 t43:2_20	20.0
54	GM2	GM2 t44:2	GM2 44:2;O3*		GM2 t44:2_20.7	20.7
55	GM3	GM3 d32:0	GM3 32:0;O2		GM3 d32:0_10.3	10.3
56	GM3	GM3 d32:1	GM3 32:1;O2	GM3 18:1;O2/14:0	GM3 d32:1_9.7	9.7
57	GM3	GM3 d33:1	GM3 33:1;O2	GM3 17:1;O2/16:0*	GM3 d33:1_10.6	10.6
58	GM3	GM3 d34:0	GM3 34:0;O2		GM3 d34:0_12.4	12.4
59	GM3	GM3 d34:1	GM3 34:1;O2	GM3 18:1;O2/16:0	GM3 d34:1_11.7	11.7
60	GM3	GM3 d34:2	GM3 34:2;O2	GM3 18:2;O2/16:0*	GM3 d34:2_9.9	9.9
61	GM3	GM3 d35:0	GM3 35:0;O2		GM3 d35:0_14	14.0
62	GM3	GM3 d35:1	GM3 35:1;O2	GM3 18:1;O2/17:0	GM3 d35:1_12.8	12.8
63	GM3	GM3 d36:0	GM3 36:0;O2		GM3 d36:0_15	15.0
64	GM3	GM3 d36:1	GM3 36:1;O2		GM3 d36:1_13	13.0
65	GM3	GM3 d36:1	GM3 36:1;O2	GM3 18:1;O2/18:0	GM3 d36:1_14.1	14.1
66	GM3	GM3 d36:2	GM3 36:2;O2	GM3 18:1;O2/18:1	GM3 d36:2_12	12.0
67	GM3	GM3 d36:2	GM3 36:2;O2	GM3 18:2;O2/18:0*	GM3 d36:2_12	12.0
68	GM3	GM3 d38:0	GM3 38:0;O2	GM3 16:0;O2/22:0*	GM3 d38:0_17.8	17.8
69	GM3	GM3 d38:1	GM3 38:1;O2		GM3 d38:1_15.3	15.3
70	GM3	GM3 d38:1	GM3 38:1;O2	GM3 18:1;O2/20:0	GM3 d38:1_16.9	16.9
71	GM3	GM3 d38:2	GM3 38:2;O2		GM3 d38:2_14.1	14.1
72	GM3	GM3 d39:1	GM3 39:1;O2	GM3 17:1;O2/22:0*	GM3 d39:1_18.4	18.4
73	GM3	GM3 d40:0	GM3 40:0;O2		GM3 d40:0_20.1	20.1
74	GM3	GM3 d40:1	GM3 40:1;O2	GM3 18:1;O2/22:0	GM3 d40:1_19.6	19.6
75	GM3	GM3 d40:2	GM3 40:2;O2	GM3 16:1;O2/24:1*	GM3 d40:2_16.7	16.7
76	GM3	GM3 d40:2	GM3 40:2;O2	GM3 18:2;O2/22:0*	GM3 d40:2_17.3	17.3
77	GM3	GM3 d41:1	GM3 41:1;O2	GM3 18:1;O2/23:0	GM3 d41:1_20.4	20.4
78	GM3	GM3 d41:2	GM3 41:2;O2	GM3 18:1;O2/23:1*	GM3 d41:2_18	18.0
79	GM3	GM3 d41:2	GM3 41:2;O2	GM3 17:1;O2/24:1*	GM3 d41:2_18	18.0
80	GM3	GM3 d41:2	GM3 41:2;O2*		GM3 d41:2_18.6	18.6
81	GM3	GM3 d42:1	GM3 42:1;O2	GM3 18:1;O2/24:0	GM3 d42:1_21	21.0
82	GM3	GM3 d42:2	GM3 42:2;O2	GM3 18:1;O2/24:1	GM3 d42:2_19.4	19.4
83	GM3	GM3 d42:2	GM3 42:2;O2	GM3 18:2;O2/24:0*	GM3 d42:2_20	20.0
84	GM3	GM3 d43:1	GM3 43:1;O2	GM3 18:2;O2/24:0*	GM3 d42:2_20	21.4
85	GM3	GM3 d43:1	GM3 43:1;O2	GM3 19:1;O2/24:0*	GM3 d43:1_21.4	21.4

86	GM3	GM3 d43:2	GM3 43:2;O2*		GM3 d43:2_19.9	19.9
87	GM3	GM3 d43:2	GM3 43:2;O2	GM3 18:1;O2/25:1*	GM3 d43:2_20.3	20.3
88	GM3	GM3 d43:2	GM3 43:2;O2	GM3 19:1;O2/24:1*	GM3 d43:2_20.3	20.3
89	GM3	GM3 d43:2	GM3 43:2;O2	GM3 20:2;O2/23:0*	GM3 d43:2_20.3	20.3
90	GM3	GM3 d44:1	GM3 44:1;O2	GM3 18:1;O2/26:0	GM3 d44:1_21.7	21.7
91	GM3	GM3 d44:2	GM3 44:2;O2		GM3 d44:2_20.6	20.6
92	GM3	GM3 d44:2	GM3 44:2;O2	GM3 18:1;O2/26:1	GM3 d44:2_20.9	20.9
93	GM3	GM3 d45:2	GM3 45:2;O2*		GM3 d45:2_21.2	21.2
94	GM3	GM3 d46:2	GM3 46:2;O2		GM3 d46:2_21.6	21.6
95	GM3	GM3 t32:1	GM3 32:1;O3		GM3 t32:1_9.2	9.2
96	GM3	GM3 t33:1	GM3 33:1;O3*		GM3 t33:1_10.1	10.1
97	GM3	GM3 t34:0	GM3 34:0;O3		GM3 t34:0_11.8	11.8
98	GM3	GM3 t34:1	GM3 34:1;O3	GM3 18:1;O3/16:0	GM3 t34:1_11.1	11.1
99	GM3	GM3 t34:2	GM3 34:2;O3	GM3 18:2;O3/16:0	GM3 t34:2_9.4	9.4
100	GM3	GM3 t35:1	GM3 35:1;O3*		GM3 t35:1_12.2	12.2
101	GM3	GM3 t36:0	GM3 36:0;O3		GM3 t36:0_12.9	12.9
102	GM3	GM3 t36:1	GM3 36:1;O3		GM3 t36:1_13.4	13.4
103	GM3	GM3 t36:2	GM3 36:2;O3		GM3 t36:2_11.3	11.3
104	GM3	GM3 t38:2	GM3 38:2;O3		GM3 t38:2_13.8	13.8
105	GM3	GM3 t40:0	GM3 40:0;O3		GM3 t40:0_17.9	17.9
106	GM3	GM3 t40:0	GM3 40:0;O3		GM3 t40:0_18.4	18.4
107	GM3	GM3 t40:1	GM3 40:1;O3		GM3 t40:1_15.6	15.6
108	GM3	GM3 t40:2	GM3 40:2;O3		GM3 t40:2_12.9	12.9
109	GM3	GM3 t41:0	GM3 41:0;O3		GM3 t41:0_19.7	19.7
110	GM3	GM3 t41:1	GM3 41:1;O3*		GM3 t41:1_16.9	16.9
111	GM3	GM3 t42:1	GM3 42:1;O3	GM3 18:0;O3/24:1	GM3 t42:1_18.2	18.2
112	GM3	GM3 t42:1	GM3 42:1;O3	GM3 19:1;O3/23:1*	GM3 t42:1_18.2	18.2
113	GM3	GM3 t42:2	GM3 42:2;O3	GM3 19:0;O3/23:2*	GM3 t42:2_15.4	15.4
114	GM3	GM3 t42:2	GM3 42:2;O3*		GM3 t42:2_15.8	15.8
115	GM3	GM3 t44:1	GM3 44:1;O3		GM3 t44:1_20.4	20.4
116	GM3	GM3 t44:2	GM3 44:2;O3*		GM3 t44:2_18.2	18.2

Extended Methods

Standards and solvents

All solvents were LC-MS grade and ordered at Fisher Scientific (Vienna, Austria), VWR International (Vienna, Austria) or Sigma Aldrich (Vienna, Austria). Ganglioside standards were from Cayman Chemical (GD2, GM4, Ann Abor, USA) or Avanti Polar Lipids, Inc. (GM3 bovine milk, total ganglioside extract (TGE) from porcine brain, Alabaster, Alabama, USA) and were weighed and dissolved in an appropriate solvent (IPA/H_2O (65%/35%, v/v)). Deuterated d5 GM1 18:1;O2/18:0 standard was purchased from Avanti Polar Lipids, Inc. (Alabaster, Alabama, USA) and used as an internal standard for further analysis. All standards were measured and manually inspected for reoccurring fragmentation pattern (Freestyle 1.8 SP2 and LDA version 2.8.3)[4,5].

Ganglioside extraction

An adapted SIMPLEX protocol[6] was used to extract gangliosides as described previously[7] since it enables the simultaneous collection of lipids (upper phase), metabolites (lower phase), and the protein pellet (see Figure 2a). In short, MSC and differentiated cell lineages (adipocytes, chondrocytes, and osteocytes) were quenched directly on the 6-well plate before storage at -80 °C until sample preparation. Deuterated internal standards were added directly to the samples to compensate for losses during extraction. Harvesting of adherent cells (~$2*10^5$ cells/well) was performed using a cell scraper. Subsequent extraction was accomplished using a mixture of cold methanol, methyl-tert-butyl ether (MTBE), and ten mM ammonium formate. Five replicates/conditions, as well as four medium blanks (control), were prepared. The lipid fractions were collected, dried, and reconstituted in IPA/H_2O (65%/35%, v/v). To determine the protein concentration, the BCA assay (Pierce kit, Thermo Fisher) was used, corresponding values can be found in Table S4.

Table S4: Protein content. Detected protein levels in all sample replicates using the BCA kit

Variant	Replicate	Protein concentration [µg/mL]
ADIPO	1	57.2
ADIPO	2	62.0
ADIPO	3	52.5
ADIPO	4	64.4
ADIPO	5	73.9
CHONDRO	1	573.0
CHONDRO	2	601.6
CHONDRO	3	496.7
CHONDRO	4	525.3
CHONDRO	5	513.4
OSTEO	1	538.9
OSTEO	2	650.9
OSTEO	3	634.2
OSTEO	4	596.1
OSTEO	5	701.0

MSC	1	48.5
MSC	2	31.8
MSC	3	50.9
MSC	4	53.2
MSC	5	62.8

Ganglioside profiling with RP-HRMS^n

A tailored ganglioside method was developed using a Vanquish Horizon UHPLC system coupled via heated electrospray-ionization (HESI) to a high-field Orbitrap ID-X™ Tribrid™ mass spectrometer (both from Thermo Fisher Scientific). RP chromatography was accomplished using an Acquity HSS T3 (2.1 mm × 150 mm, 1.8 µm, Waters) column with a VanGuard pre-column (2.1 mm × 5 mm, 100 Å, 1.8 µm). The column temperature was set to 40 °C, the flow rate was set to 0.25 mL min$^{-1,}$ and the injection volume was set to 5 µL. The injector needle was flushed with 75% isopropanol (IPA) and 1% formic acid (FA) in between the injections. Acetonitrile (ACN)/H2O (3:2, v/v) was used as solvent A and IPA/ACN (9:1, v/v) as solvent B, both containing 0.1% formic acid and 10 mM ammonium formate. A gradient of 30 minutes under following conditions was applied: 0.0-2.0 min 30% B, 2.0-3.0 min ramp to 55% B, 3.0-17.0 min ramp to 67% B, 17.0-22.0 min ramp to 100 %, 22.0-26.0 min 100% B, 26.0 min fast switch to 30% B and 26.0-30.0 equilibration at starting conditions (30% B).

The ESI source parameters were as follows: 3.5 kV (positive ion mode) and 3.0 kV (negative ion mode), sheath gas 40, auxiliary gas 8, sweep gas 1, capillary temperature (ion transfer tube temperature) 275 °C, auxiliary gas heater (vaporizer temperature) 350 °C, radio frequency (RF) level 45%. Positive and negative ionization mode data were acquired in separate runs.

Spectral data were acquired in profile mode. For full MS runs, a mass range of *m/z* 500-2,000 at a resolution of 120,000 was selected. The automatic gain control (AGC) target was set to standard, the maximum injection time (MIT) was 100 ms. MS2 and MS3 scans were performed with data-dependent acquisition (DDA). For MS2 scanning, a top 5 method with a resolution of 15,000 was applied. The normalized collision energy (NCE) was set to 23 (+) and 27 (-) (HCD activation), respectively, the isolation window to *m/z* 1.5, the AGC target to standard, and the MIT to 60 ms. The dynamic exclusion of triggered *m/z* was set to 5 s, and a ganglioside-specific inclusion list was implemented. For MS3 spectra, the mass range was reduced to *m/z* 300-800 to gain fragments of the ceramide moieties (LCB, FA) to elucidate the molecular lipid species composition. The Ion Trap was selected as a mass analyzer with a fixed collision energy of 30% (CID activation), with 10 ms activation time and an activation Q of 0.25. The Ion Trap scan rate was set to rapid, the isolation window to *m/z* 1.5 (MS1) and 2.0 (MS2), respectively, the AGC target was set to standard and MIT to automatic. The MS3 scans in the Ion trap were parallelizable with MS1 and MS2 scans in the Orbitrap, increasing information content and saving time (**Figure 2b**).

Deep ganglioside and lipid profiling (deep scans) were performed on sequential automated exclusion lists (including blank subtraction) enabled by AcquireX data acquisition software within the Orbitrap ID-X™ Tribrid™ mass spectrometer.

Figure S7: MS workflow. Scan parameters of the developed MS^n workflow on the ID-X in positive (left) and negative (right) ionization mode. In negative mode an additional sialic acid fragment specific mass trigger of m/z = 290.0876 was set as filter for the MS3 scan in the ion trap, as the fragment was observed in all investigated ganglioside classes.

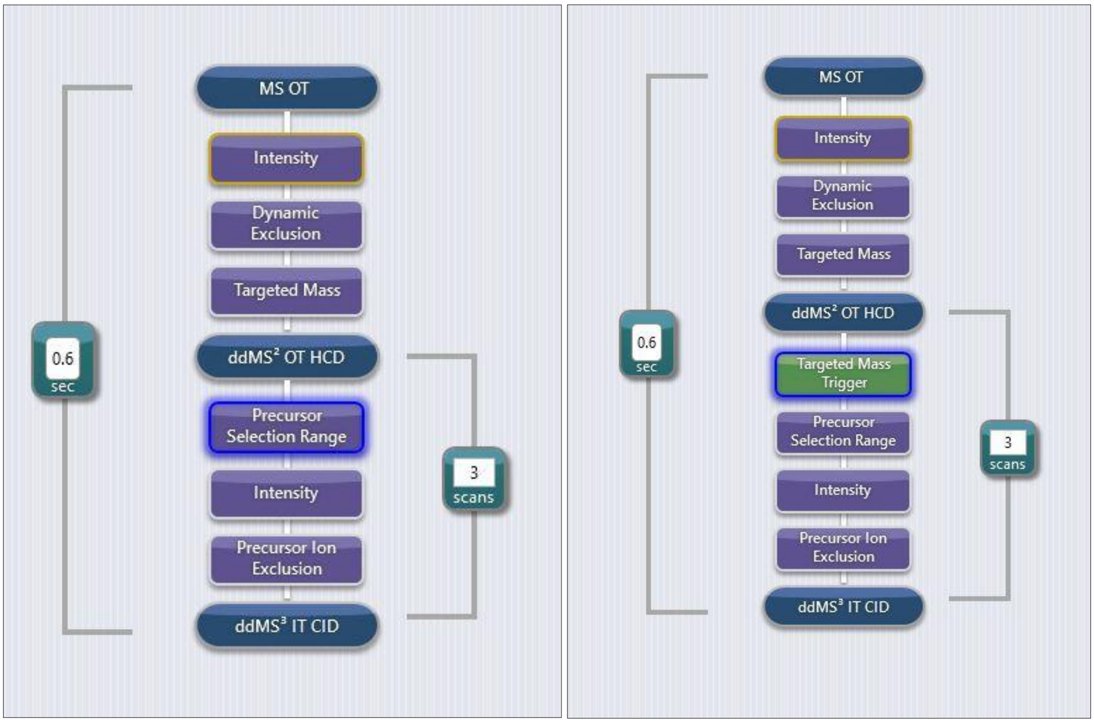

<u>Data evaluation</u>

LDA

The ganglioside assignment was performed using the LDA (version 2.8.3)[4,5], the *OrbiTrap_IDX_heavy* settings were optimized and can be found in the provided LDA version http://genome.tugraz.at/lda2 including necessary decision rule sets and corresponding mass lists. For the development of the implemented decision rules (fragRules\OrbiTrap_IDX_heavy in the LDA software) available standards (GM3, GM4, GD2, TGE, all around 5 µM in IPA/H2O, 65%/35% (v/v)) were measured with the developed ID-X workflow presented above and processed with the LDA software including isotopic quantitation of 2 isotopes. Using the statistical analysis within the LDA software, analyzed standards were manually inspected (Show hits with different RT separately: 0.3 min, box ticked) for reoccurring fragmentation pattern. For each ganglioside class the list of possible fragments

was further extended with at least one class-specific fragment (fragRules, mandatory=true), to minimize false positive annotations. Further, the parameter SingleChainIdentification was set to false, therefore the molecular lipid species level can only be assigned when both chain parts (LCB and FA) are detected. The presented LDA version furthermore includes optimized peak integration for heavy masses (compared to smaller lipids) as well as adapted mass accuracy tolerances for the ion trap. 10 different adducts are currently considered including [M-3H]$^{3-}$, [M-2H]$^{2-}$, [M+HCOO]$^{-}$, [M+Na-2H]$^{-}$, [M-H]$^{-}$, [M+H]$^{+}$, [M+NH4]$^{+}$, [M+Na]$^{+}$, [M+2H]$^{2+}$, and [M+3H]$^{3+}$. This list can easily be extended by the user, if necessary. After optimizing the LDA software with standards, measurement of the samples as well as negative controls, blanks and qc files was performed. "Batch Quantitation" was performed at University of Graz using default parameters and "OrbiTrap_IDX_heavy" settings with developed ganglioside specific fragRules. Subsequently positive and negative ion mode were processed together at the "Statistical Analysis" tab with a RT tolerance of 0.3 min and exported using the rdb export option.

Table S5: Fragmentation rule example. Example of GM3 [M-H]$^{-}$ as included in the LDA software. In the [GENERAL] section, various general parameters can be defined. Details about these parameters can be found in the LDA user manual. In this example, "SingleChainIdentification" is set to false, requesting LDA to assign molecular lipid species level only if both LCB and FA fragments are detected. In the "[HEAD] FRAGMENTS" section, GM3 specific fragments are specified, including neutral (glycan) losses. In the "[CHAIN] FRAGMENTS" section, fragments of the ceramide part are defined. "mandatory=true" fragments must be detected for an annotation at the corresponding structural level (marked in grey). The developed fragrules are supplied with the software and allow successful annotation of 12 different ganglioside classes and hundreds of potential combinations. The LDA software allows user-specific adaptions based on observed fragmentation patterns. As such it offers automated and high-quality annotations, which is not limited to ganglioside profiling.

```
[GENERAL]
AmountOfChains=2
AmountOfLCBs=1
ChainLibrary=fattyAcidChains.xlsx
LCBLibrary=dLCB.xlsx
CAtomsFromName=\D*(\d+):\d+
DoubleBondsFromName=\D*\d+:(\d+)
ChainCutoff=50%
FaHydroxylationRange=0-1
LcbHydroxylationRange=2-3
RetentionTimePostprocessing=true
SingleChainIdentification=false
[HEAD]
!FRAGMENTS
Name=NeuAcFrag_87       Formula=C3H3O3       Charge=1   MSLevel=2   mandatory=false
Name=NeuAc-H2O_272      Formula=C11H14NO7    Charge=1   MSLevel=2   mandatory=false
Name=Hex_161            Formula=C6H9O5       Charge=1   MSLevel=2   mandatory=false
Name=Hex-O_179          Formula=C6H11O6      Charge=1   MSLevel=2   mandatory=false
Name=NeuAc_290          Formula=C11H16NO8    Charge=1   MSLevel=2   mandatory=true
```

Name=NeuAc-O_308	Formula=C11H18NO9	Charge=1	MSLevel=2	mandatory=false	
Name=Hex-Hex_323	Formula=C12H19O10	Charge=1	MSLevel=2	mandatory=false	
Name=Hex-Hex-O_341	Formula=C12H21O11	Charge=1	MSLevel=2	mandatory=false	
Name=NeuAc-Hex_452	Formula=C17H26NO13	Charge=1	MSLevel=2	mandatory=false	
Name=NeuAc-Hex-O_470	Formula=C17H28NO14	Charge=1	MSLevel=2	mandatory=false	
Name=NeuAc-Hex-Hex_614	Formula=C23H36NO18	Charge=1	MSLevel=2	mandatory=false	
Name=NeuAc-Hex-Hex-O_632	Formula=C23H38NO19	Charge=1	MSLevel=2	mandatory=false	
Name=NeuAcFrag_87_MS3	Formula=C3H3O3	Charge=1	MSLevel=3	mandatory=false	
Name=NeuAc-H2O_272_MS3	Formula=C11H14NO7	Charge=1	MSLevel=3	mandatory=false	
Name=Hex_161_MS3	Formula=C6H9O5	Charge=1	MSLevel=3	mandatory=false	
Name=Hex-O_179_MS3	Formula=C6H11O6	Charge=1	MSLevel=3	mandatory=false	
Name=NeuAc_290_MS3	Formula=C11H16NO8	Charge=1	MSLevel=3	mandatory=false	
Name=NeuAc-O_308_MS3	Formula=C11H18NO9	Charge=1	MSLevel=3	mandatory=false	
Name=Hex-Hex_323_MS3	Formula=C12H19O10	Charge=1	MSLevel=3	mandatory=false	
Name=Hex-Hex-O_341_MS3	Formula=C12H21O11	Charge=1	MSLevel=3	mandatory=false	
Name=NeuAc-Hex_452_MS3	Formula=C17H26NO13	Charge=1	MSLevel=3	mandatory=false	
Name=NeuAc-Hex-O_470_MS3	Formula=C17H28NO14	Charge=1	MSLevel=3	mandatory=false	
Name=NeuAc-Hex-Hex_614_MS3	Formula=C23H36NO18	Charge=1	MSLevel=3	mandatory=false	
Name=NeuAc-Hex-Hex-O_632_MS3	Formula=C23H38NO19	Charge=1	MSLevel=3	mandatory=false	
Name=Precursor	Formula=$PRECURSOR	Charge=1	MSLevel=2	mandatory=false	
Name=M-Hex_M-162	Formula=$PRECURSOR-C6H10O5	Charge=1	MSLevel=2	mandatory=false	
Name=M-Hex-O_M-180	Formula=$PRECURSOR-C6H12O6	Charge=1	MSLevel=2	mandatory=false	
Name=M-NeuAc_M-291	Formula=$PRECURSOR-C11H17NO8	Charge=1	MSLevel=2	mandatory=false	
Name=M-NeuAc-O_M-309	Formula=$PRECURSOR-C11H19NO9	Charge=1	MSLevel=2	mandatory=false	
Name=M-Hex-Hex_M-324	Formula=$PRECURSOR-C12H20O10	Charge=1	MSLevel=2	mandatory=false	
Name=M-Hex-Hex-O_M-342	Formula=$PRECURSOR-C12H22O11	Charge=1	MSLevel=2	mandatory=false	
Name=M-NeuAc-Hex_M-453	Formula=$PRECURSOR-C17H27NO13	Charge=1	MSLevel=2	mandatory=false	
Name=M-NeuAc-Hex-O_M-471	Formula=$PRECURSOR-C17H29NO14	Charge=1	MSLevel=2	mandatory=false	
Name=M-NeuAc-Hex-Hex_M-615	Formula=$PRECURSOR-C23H37NO18	Charge=1	MSLevel=2	mandatory=false	
Name=M-NeuAc-Hex-Hex-O_M-633	Formula=$PRECURSOR-C23H39NO19	Charge=1	MSLevel=2	mandatory=false	
Name=M-Hex_M-162_MS3	Formula=$PRECURSOR-C6H10O5	Charge=1	MSLevel=3	mandatory=false	
Name=M-Hex-O_M-180_MS3	Formula=$PRECURSOR-C6H12O6	Charge=1	MSLevel=3	mandatory=false	
Name=M-NeuAc_M-291_MS3	Formula=$PRECURSOR-C11H17NO8	Charge=1	MSLevel=3	mandatory=false	
Name=M-NeuAc-O_M-309_MS3	Formula=$PRECURSOR-C11H19NO9	Charge=1	MSLevel=3	mandatory=false	
Name=M-Hex-Hex_M-324_MS3	Formula=$PRECURSOR-C12H20O10	Charge=1	MSLevel=3	mandatory=false	
Name=M-Hex-Hex-O_M-342_MS3	Formula=$PRECURSOR-C12H22O11	Charge=1	MSLevel=3	mandatory=false	
Name=M-NeuAc-Hex_M-453_MS3	Formula=$PRECURSOR-C17H27NO13	Charge=1	MSLevel=3	mandatory=false	
Name=M-NeuAc-Hex-O_M-471_MS3	Formula=$PRECURSOR-C17H29NO14	Charge=1	MSLevel=3	mandatory=false	
Name=M-NeuAc-Hex-Hex_M-615_MS3	Formula=$PRECURSOR-C23H37NO18	Charge=1	MSLevel=3	mandatory=false	
Name=M-NeuAc-Hex-Hex-O_M-633_MS3	Formula=$PRECURSOR-C23H39NO19	Charge=1	MSLevel=3	mandatory=false	
**[CHAINS]**					
**!FRAGMENTS**					
Name=FA_FA	Formula=$CHAIN	Charge=1	MSLevel=2	mandatory=false	
Name=FA-H_FA-1	Formula=$CHAIN-H	Charge=1	MSLevel=2	mandatory=false	
Name=FA-H2O_FA-18	Formula=$CHAIN-H2O	Charge=1	MSLevel=2	mandatory=false	
Name=FA-H3O_FA-19	Formula=$CHAIN-H3O	Charge=1	MSLevel=2	mandatory=false	
Name=FA-H4O_FA-20	Formula=$CHAIN-H4O	Charge=1	MSLevel=2	mandatory=false	
Name=LCB-H2O_LCB-18	Formula=$LCB-H3O	Charge=1	MSLevel=2	mandatory=false	
Name=LCB-2H2O_LCB-36	Formula=$LCB-H5O2	Charge=1	MSLevel=2	mandatory=false	
Name=LCB-H6ON_LCB-36	Formula=$LCB-H6ON	Charge=1	MSLevel=2	mandatory=false	oh=2
Name=LCB-CH4O2_LCB-48	Formula=$LCB-CH5O2	Charge=1	MSLevel=2	mandatory=false	
Name=LCB-H6ON_LCB-50	Formula=$LCB-CH8ON	Charge=1	MSLevel=2	mandatory=false	oh=3
Name=FA_FA_MS3	Formula=$CHAIN	Charge=1	MSLevel=3	mandatory=false	
Name=FA-H_FA-1_MS3	Formula=$CHAIN-H	Charge=1	MSLevel=3	mandatory=false	
Name=FA-H2O_FA-18_MS3	Formula=$CHAIN-H2O	Charge=1	MSLevel=3	mandatory=false	
Name=FA-H3O_FA-19_MS3	Formula=$CHAIN-H3O	Charge=1	MSLevel=3	mandatory=false	
Name=FA-H4O_FA-20_MS3	Formula=$CHAIN-H4O	Charge=1	MSLevel=3	mandatory=false	
Name=LCB-H2O_LCB-18_MS3	Formula=$LCB-H3O	Charge=1	MSLevel=3	mandatory=false	

Name=LCB-2H2O_LCB-36_MS3	Formula=$LCB-H5O2	Charge=1	MSLevel=3	mandatory=false	
Name=LCB-H6ON_LCB-36_MS3	Formula=$LCB-H6ON	Charge=1	MSLevel=3	mandatory=false	oh=2
Name=LCB-CH4O2_LCB-48_MS3	Formula=$LCB-CH5O2	Charge=1	MSLevel=3	mandatory=false	
Name=LCB-H6ON_LCB-50_MS3	Formula=$LCB-CH8ON	Charge=1	MSLevel=3	mandatory=false	oh=3

Skyline

Areas of the internal standard (d5 GM1 36:1;O2) was received from Skyline (version 21.2), including the same (deuterated) adducts as within the LDA, and subsequently exported as a csv file. LDA (ganglioside annotations), Skyline (internal standard), and BCA (protein content) results were then evaluated using R Studio (version 4.2.1).

Figure S8: Skyline for internal standards. Integration of internal standard in different sample groups using Skyline.

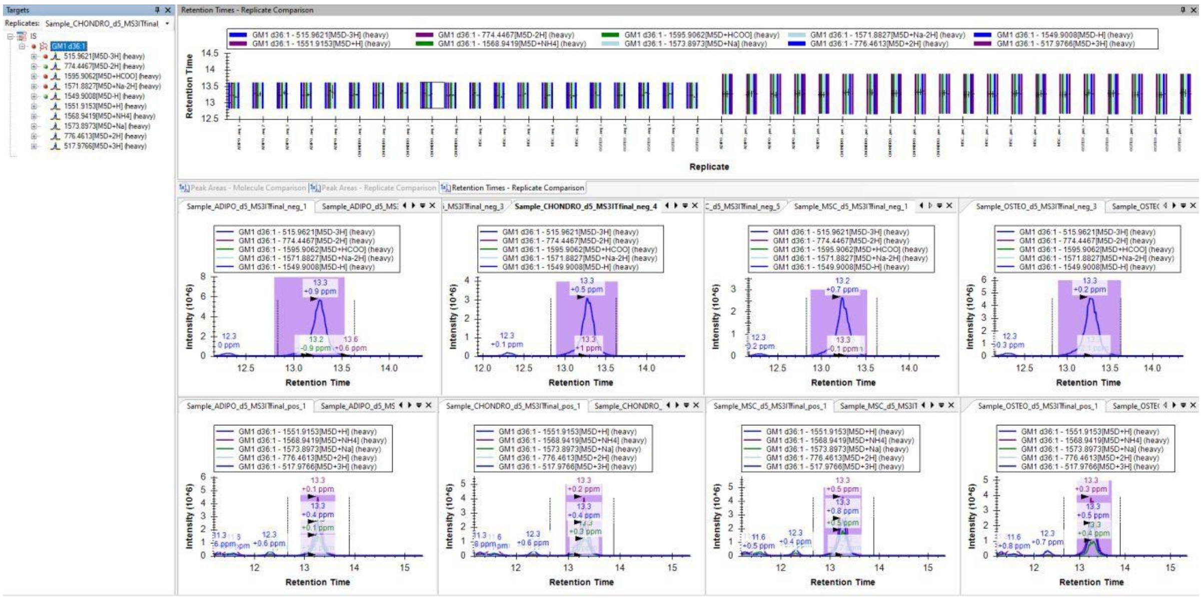

R Studio and MetaboAnalyst

All LDA analysis results in both polarities were processed together (Statistical Analysis) by retention time alignment (RT group). The results were subsequently exported using the rdb export option. Areas of internal standards were received from Skyline (version 21.2) and exported as a csv file. LDA (ganglioside annotations), Skyline (internal standard), and BCA (protein content) results were then evaluated using R Studio (version 4.2.1). Unique ganglioside hits (IDs) were curated using the lipid species as well as the retention time group information of the LDA export. Several quality control filters were applied, including (1) retention time filter (2-26 min), (2) ppm filter (max. 5 ppm), (3) area threshold filter (min 30,000), (4) single annotation filter (at least 3 detections over all files), and (5)

MS2 filter (at least one MS2 spectrum in standard or sample file). Moreover, only trihydroxylated species with a corresponding dihydroxylated species where considered. The impact of each filter can be seen in **Table S6**. Retention time matching of adducts in both polarities were performed based on the generated ID. Sum of adducts were calculated for each polarity and each ID. All annotations were normalized to the sum of adducts pertaining to the internal standard (IS d5 GM1 36:1;O2) in the respective polarity. The lipid species' concentration was estimated via one-point calibration using the known concentration of the internal standards. Subsequently, normalization to the protein content was performed, followed by calculation of mean and standard deviation for sample replicates. As a final filter, the ECN models[8] for each ganglioside class were plotted and manually filtered, whereby fitting retention times according to the ECN model, MS1 peak shape, and MS2 spectral quality were considered, resulting in 137 unique annotated and semi-quantified lipid species in the four different sample types.

In general, positive ion mode data was predominantly used to corroborate the annotations detected in negative ion mode (matching RTs) and where possible, for obtaining annotations at the molecular lipid species level. Quantities presented in figures and tables are the sum of adduct abundances detected in negative ion mode, as this ion mode provided better coverage with respect to the number of detected species.

Figure S9: ECN model at the example of identified GM2 gangliosides. Hydroxylation state is indicated with shapes (dihydroxylated = circle, trihydroxylated = triangle), number of double bonds (DB = 0, 1, 2) is indicated with different blue colors. Linear correlation between RT and carbon number can be seen. Moreover, the effect of decreasing RT with increasing DB number (with same carbon number) could be observed. Trihydroxylated species eluted before the dihydroxylated species. In several cases, isomeric species could be chromatographically resolved providing more than one distinct retention time for the same lipid species (GM2 d42:2_19.1 and GM2 d42:2_19.8). "species" corresponds to the identified lipid species with the corresponding RT attached (in minutes).

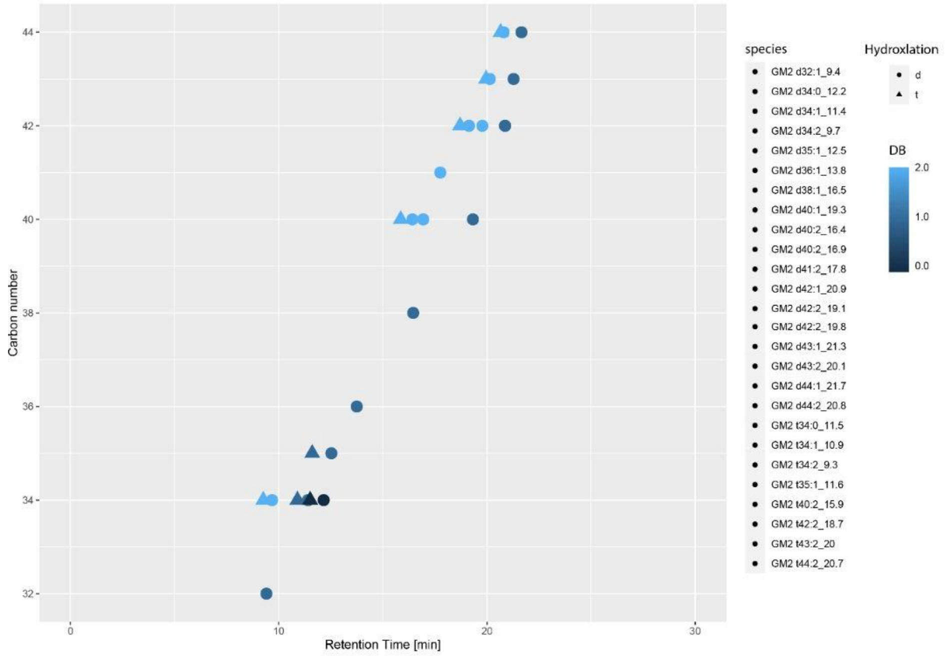

.

MetaboAnalyst result filtering: For statistical analysis the subset of 137 annotated hits in the MSCs and differentiation samples were exported to MetaboAnalyst 5.0 using the “Statistical Analysis [one factor]”. “Data filtering” was set to “None (less than 5000 features)”, “Sample normalization” and “Data transformation” were set to “none”, “Data scaling” to “Auto-scaling”. One-way ANOVA & post-hoc Tests was carried out using an adjusted p-value (FDR) cutoff of 0.05 and Tukey´s HSD as Post-hoc analysis. PCA analysis was performed using standard settings. All data were downloaded, reimported to R Studio and combined with the original data table for further data processing and visualization (R libraries: data.table, viridis, tidyverse, plotly, ggforce, patchwork, and pheatmap). For all significantly regulated annotations (p-value below 0.05) molecular lipid species levels where assigned, whenever the spectra quality allowed a clear assignment (**Table S3**). Notably, over 60% of the assigned molecular lipid species could only be identified by respective MS3 scans, emphasizing the power of the developed MS^n method.

Table S6: Applied filters during data processing. Filters applied using R Studio. Class = Lipid class, Species = unique lipid species, ID_1 = unique lipid species by RT group (potential isomeric species), Sample = MSC, adipo, chondro or osteo, stds = external standards, QC = pooled samples and total ganglioside extract measured several time over the sequence, d, t species = di- or trihydroxylated species.

Filter	Data included	Class	Species	ID_1	Comment
0. No filter	Samples, stds, QCs, Blank	12	1342	8659	
1. RT filter	Samples, stds, QCs, Blank	12	1302	6309	2-26 min
2. Ppm filter	Samples, stds, QCs, Blank	12	1286	5674	Max 5 ppm
3. Area threshold	Samples, stds, QCs, Blank	12	872	1993	> 30,000
4. Single hits	Samples, stds, QCs, Blank	12	485	776	Min 3 detections (in all files)
5. MS2 filter	Samples, stds, QCs, Blank	10	188	254	Min 1 MS2 in sample or std

6. Check MS3 scans	Check only		91 (48.4%)	113 (44.4%)	See below
7. d, t species	Samples, stds, QCs, Blank	10	165	227	Keep only t species with corresponding d species (RT not considered)
8. ECN model	Samples, stds, QCs, Blank	9	140	171	Manual data curation
9. Quantification	Samples only	6	115	137	Sum of adducts, mean, sd
10. Significant hits	Samples only	6	97	110	Export (MetaboAnalyst) and reimport
11. Potential markers	Samples only	6		78	Significantly up/downregulation for one sample group (marker)

Figure S10: Effect of applied filters to different sample types. **a** Summed area (not normalized) in different sample types before filters are applied. **b** Summed area (not normalized) in different sample types after filters are applied. Sufficient filtering of false positive hits leads to almost no background. Blank = solvent blank and extraction blank, ctrl = negative control of sample, ESTD = external standards, QC = pooled sample, Sample = MSC, adipo, chondro and osteo.

a

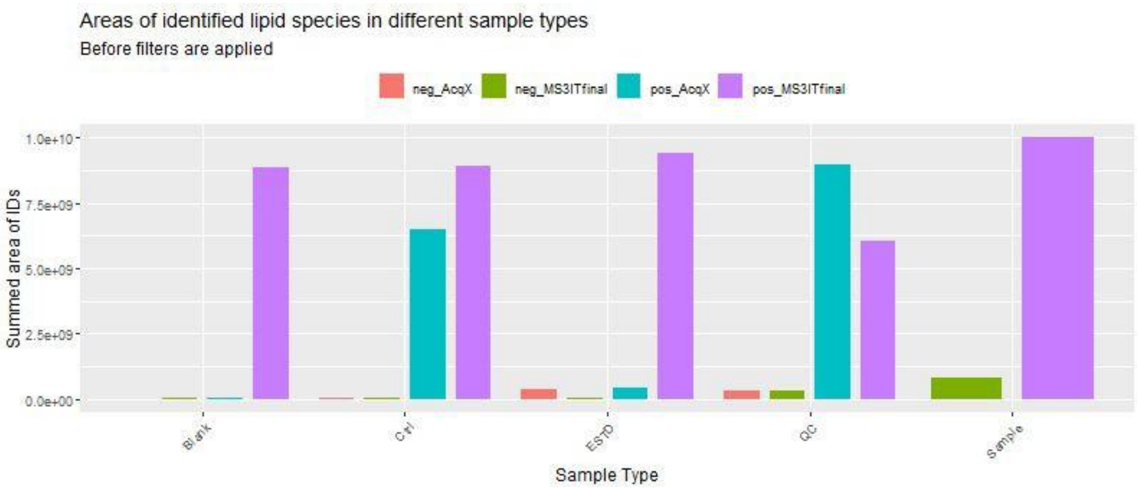

b

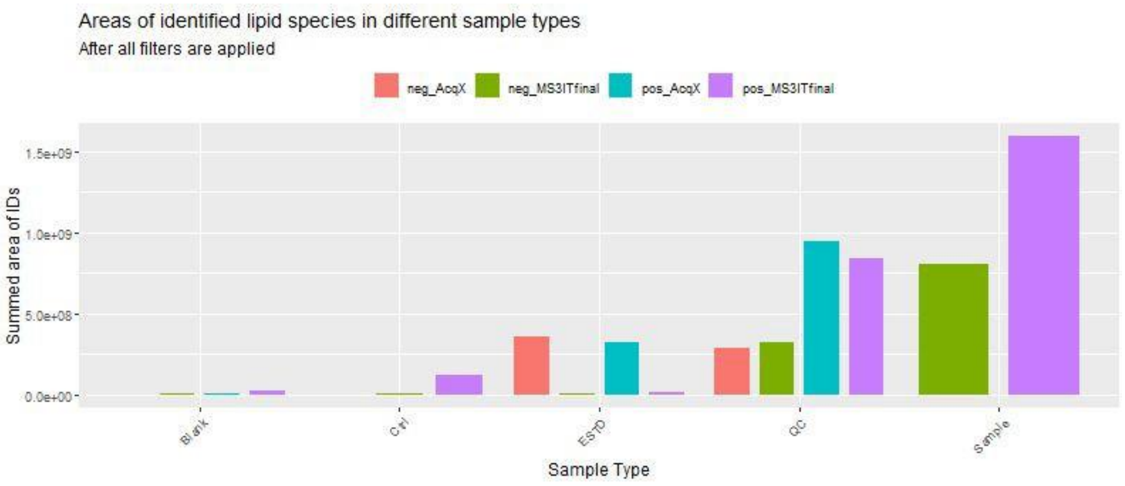

References

(1) Sud, M.; Fahy, E.; Cotter, D.; Brown, A.; Dennis, E. A.; Glass, C. K.; Merrill, A. H.; Murphy, R. C.; Raetz, C. R. H.; Russell, D. W.; Subramaniam, S. LMSD: LIPID MAPS Structure Database. *Nucleic Acids Res.* **2007**, *35* (Database), D527–D532. https://doi.org/10.1093/nar/gkl838.

(2) Lipid Web Glycan series https://www.lipidmaps.org/resources/lipidweb/lipidweb_html/lipids/sphingo/oligocer/index.htm.

(3) Domon, B.; Costello, C. E. A Systematic Nomenclature for Carbohydrate Fragmentations in FAB-MS/MS Spectra of Glycoconjugates. *Glycoconjugate J. 1988 54* **1988**, *5* (4), 397–409. https://doi.org/10.1007/BF01049915.

(4) Hartler, J.; Trötzmüller, M.; Chitraju, C.; Spener, F.; Köfeler, H. C.; Thallinger, G. G. Lipid Data Analyzer: Unattended Identification and Quantitation of Lipids in LC-MS Data. *Bioinformatics* **2011**, *27* (4), 572–577. https://doi.org/10.1093/bioinformatics/btq699.

(5) Hartler, J.; Armando, A. M.; Trötzmüller, M.; Dennis, E. A.; Köfeler, H. C.; Quehenberger, O. Automated Annotation of Sphingolipids Including Accurate Identification of Hydroxylation Sites Using MSn Data. *Anal. Chem.* **2020**, *92* (20), 14054–14062. https://doi.org/10.1021/acs.analchem.0c03016.

(6) Coman, C.; Solari, F. A.; Hentschel, A.; Sickmann, A.; Zahedi, R. P.; Ahrends, R. Simultaneous Metabolite, Protein, Lipid Extraction (SIMPLEX): A Combinatorial Multimolecular Omics Approach for Systems Biology. *Mol. Cell. Proteomics* **2016**, *15* (4), 1435–1466. https://doi.org/10.1074/mcp.M115.053702.

(7) Rampler, E.; Egger, D.; Schoeny, H.; Rusz, M.; Pacheco, M. P.; Marino, G.; Kasper, C.; Naegele, T.; Koellensperger, G. The Power of LC-MS Based Multiomics: Exploring Adipogenic Differentiation of Human Mesenchymal Stem/Stromal Cells. *Molecules* **2019**, *24* (19), 3615. https://doi.org/10.3390/molecules24193615.

(8) Holčapek, M.; Ovčačíková, M.; Lísa, M.; Cífková, E.; Hájek, T. Continuous Comprehensive Two-Dimensional Liquid Chromatography–Electrospray Ionization Mass Spectrometry of Complex Lipidomic Samples. *Anal. Bioanal. Chem.* **2015**, *407* (17), 5033–5043. https://doi.org/10.1007/s00216-015-8528-2.
